# Fatty acid oxidation organizes mitochondrial supercomplexes to sustain astrocytic ROS and cognition

**DOI:** 10.1038/s42255-023-00835-6

**Published:** 2023-07-17

**Authors:** Brenda Morant-Ferrando, Daniel Jimenez-Blasco, Paula Alonso-Batan, Jesús Agulla, Rebeca Lapresa, Dario Garcia-Rodriguez, Sara Yunta-Sanchez, Irene Lopez-Fabuel, Emilio Fernandez, Peter Carmeliet, Angeles Almeida, Marina Garcia-Macia, Juan P. Bolaños

**Affiliations:** 1 Institute of Functional Biology and Genomics (IBFG), University of Salamanca, CSIC, Salamanca, Spain; 2 Institute of Biomedical Research of Salamanca (IBSAL), University Hospital of Salamanca, Salamanca, Spain; 3 Centre for Biomedical Investigations Network on Frailty and Ageing (CIBERFES), Madrid, Spain; 4 Laboratory of Angiogenesis and Vascular Metabolism, Vesalius Research Center, Leuven, Belgium

**Keywords:** Cell biology, Molecular medicine, Metabolism, Glial biology

## Abstract

Having direct access to brain vasculature, astrocytes can take up available blood nutrients and metabolize them to fulfil their own energy needs and deliver metabolic intermediates to local synapses^1,2^. These glial cells should be, therefore, metabolically adaptable to swap different substrates. However, in vitro and in vivo studies consistently show that astrocytes are primarily glycolytic^3–7^, suggesting glucose is their main metabolic precursor. Notably, transcriptomic data^8,9^ and in vitro^10^ studies reveal that mouse astrocytes are capable of mitochondrially oxidizing fatty acids and that they can detoxify excess neuronal-derived fatty acids in disease models^11,12^. Still, the factual metabolic advantage of fatty acid use by astrocytes and its physiological impact on higher-order cerebral functions remain unknown. Here, we show that knockout of carnitine-palmitoyl transferase-1A (CPT1A)—a key enzyme of mitochondrial fatty acid oxidation—in adult mouse astrocytes causes cognitive impairment. Mechanistically, decreased fatty acid oxidation rewired astrocytic pyruvate metabolism to facilitate electron flux through a super-assembled mitochondrial respiratory chain, resulting in attenuation of reactive oxygen species formation. Thus, astrocytes naturally metabolize fatty acids to preserve the mitochondrial respiratory chain in an energetically inefficient disassembled conformation that secures signalling reactive oxygen species and sustains cognitive performance.

## Abstract (web summary)

In this study, Morant-Ferrando, Jiménez-Blasco et al. show that fatty acid oxidation in astrocytes is necessary to maintain a specific configuration of the electron transport chain, which enables controlled production of reactive oxygen species that fine-tune neuron–glia metabolic coupling and support cognitive function.

## Main

To ascertain the expression levels of genes coding for fatty acids use in astrocytes and neurons, we performed quantitative PCR with reverse transcription (RT–qPCR) analyses, which revealed increased messenger RNA abundances in carnitine-palmitoyl transferase-1A (*Cpt1a*)—responsible for long-chain acyl-CoA entry into mitochondria^13^—and decreased acetyl-CoA carboxylase-1 (*Acc1*)—responsible for the biosynthesis of CPT1A^−^ inhibitor malonyl-CoA^14^-mRNA abundances in mouse primary astrocytes when compared with neurons (Supplementary Fig. 1a). In addition, the mRNA abundances of mitochondrial *Acc2* isoform, which is found highly enriched in oxidative tissues such as skeletal muscle and heart^15^, of long-chain acetyl-CoA dehydrogenase (*Acadl*), which catalyses the initial step of mitochondrial fatty acid oxidation, of the endoplasmic reticulum-located *Cpt1c* and of mitochondrial trifunctional protein α (*Mtpα*) that is functionally responsible for electron transfer from mitochondrial long-chain fatty acids to mitochondrial respiratory complexes I (CI) and III (CIII)^16^, were found to be higher in astrocytes versus neurons (Supplementary Fig. 1a). Although these are relative values, they are in coherence with previous observations^8–10^ suggesting that astrocytes are better equipped than neurons to mitochondrially oxidize long-chain fatty acids. To functionally sustain this statement, the oxygen consumption rate (OCR) was analysed in astrocytes and neurons using the Seahorse technology in glucose-based medium in either the absence or presence of etomoxir: a potent and irreversible inhibitor of CPT1 (ref. ^17^). As shown in Supplementary Fig. 1b, basal mitochondrial respiration was found to be roughly 1.7-fold higher in neurons when compared with astrocytes, confirming previous findings^18^. Notably, the proportion of mitochondrial OCR inhibition by etomoxir was roughly 20% in neurons and roughly 35% in astrocytes (Supplementary Fig. 1b), indicating that fatty acids are preferred mitochondrial respiratory substrates for astrocytes than neurons. Roughly 62% of astrocytic ATP-linked mitochondrial respiration was sustained by fatty acids (Supplementary Fig. 1b).

**Fig. 1:**
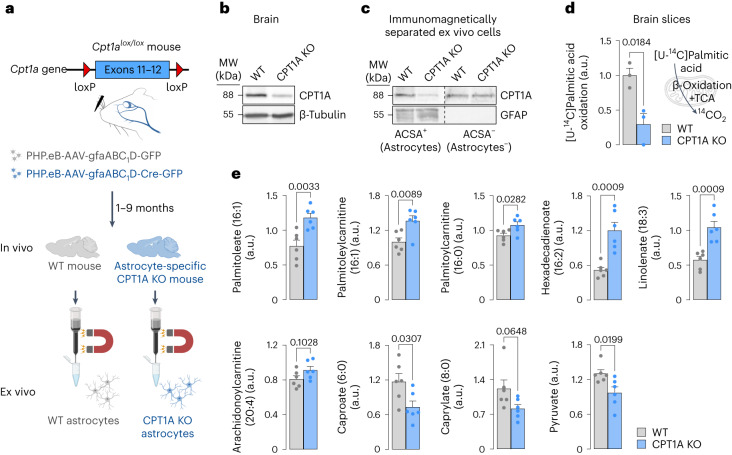
In vivo astrocyte-specific *Cpt1a* KO inhibits fatty acid oxidation and alters the metabolomics pattern in the brain. **a**, Strategy used to generate astrocyte-specific *Cpt1a* KO mice and to immunomagnetically purify CPT1A KO astrocytes from adult brain. Created with BioRender.com. **b**, Western blot against CPT1A protein in astrocyte-specific *Cpt1a* KO brain. β-Tubulin was used as a loading control; *n* = 2 mice per condition (Supplementary Fig. 1d). **c**, Western blotting against CPT1A protein in ACSA^+^ (astrocytes) and ACSA^−^ (not astrocytes) cells, immunomagnetically isolated from astrocyte-specific *Cpt1a* KO mouse brain; *n* = 2 mice per condition. GFAP was used as astrocyte enrichment and loading controls. **d**, Rate of ^14^CO_2_ production from [U-^14^C]palmitic acid in brain slices of WT and astrocyte-specific *Cpt1a* KO mice. Data are mean ± s.e.m. *P* value is indicated (*n* = 3 biologically independent samples; unpaired Student’s *t*-test, two-sided). **e**, Concentrations of a selection of metabolites altered in the metabolomics study of the brain samples from astrocyte-specific *Cpt1a* KO when compared with WT mice. Data are mean ± s.e.m. *P* values are indicated (*n* = 6 mice per condition; unpaired Student’s *t*-test, two-sided). a.u., arbitrary units. Source data

To investigate the metabolic advantage of mitochondrial fatty acid oxidation in astrocytes in vivo, otherwise overcoming the potential drawbacks of pharmacological inhibitors, we genetically engineered an astrocyte-specific *Cpt1a* knockout (KO) mouse model. To do so, 2-month-old *Cpt1a*^*lox/lox*^ mice^19^ were intravenously injected, via the retro-orbital sinus^20^, with PHP.eB serotype adeno-associated virus (AAV) particles expressing Cre recombinase governed by the astrocytic-specific glial-fibrillary acidic protein (GFAP) short-promoter (PHP.eB-AAV-gfaABC_1_D-Cre-GFP) (Fig. 1a). This treatment was efficient, as judged by the wide expression of green fluorescent protein (GFP) across the brain (Supplementary Fig. 1c). Controls (wild-type, WT) were *Cpt1a*^*lox/lox*^ mice that received equivalent doses of the same virus particles, except that they lacked Cre recombinase, and all mice were analysed after 1–9 months (Fig. 1a). As shown in Fig. 1b (Supplementary Fig. 1d), PHP.eB-AAV-gfaABC_1_D-Cre-GFP treatment caused a significant reduction in brain CPT1A protein abundance. Given that the brain contains other cell types besides astrocytes, we also analysed CPT1A abundance in ex vivo astrocytes immunomagnetically isolated from the brain of the adult CPT1A KO mice (Fig. 1a), which revealed CPT1A abolishment specifically in the astrocyte-positive (ACSA^+^) fraction, but not in the astrocyte-negative (ACSA^−^) fraction (Fig. 1c). To ascertain the functional efficacy of CPT1A KO, ex vivo freshly isolated brain slices from adult mice were incubated with [U-^14^C]palmitic acid to assess the rate of ^14^CO_2_ production as an index of fatty acid oxidation flux. As shown in Fig. 1d, oxidation flux in the brain was significantly reduced by roughly 75% in CPT1A KO when compared with WT mice. To explain whether loss of astrocytic CPT1A altered other pathways of brain metabolism, we performed untargeted metabolomics in brain samples. As depicted in the volcano plot (Supplementary Fig. 1e) and in the heatmap (Supplementary Fig. 1f), we found 17 metabolites significantly decreased and 43 metabolites significantly increased in the brain of the astrocyte-specific CPT1A KO mice. The results revealed increased abundance in long-chain fatty acids and long-chain acyl-carnitine derivatives, and decreased abundance in short-chain fatty acids (Fig. 1e), suggesting decreased long-chain and increased short-chain fatty acid use. Notably, pyruvate concentration was significantly decreased by roughly 26% in the brain of astrocyte-specific CPT1A KO mice (Fig. 1e), suggesting an alteration in the metabolism of this glycolytic-end product intermediate.

**Fig. 2:**
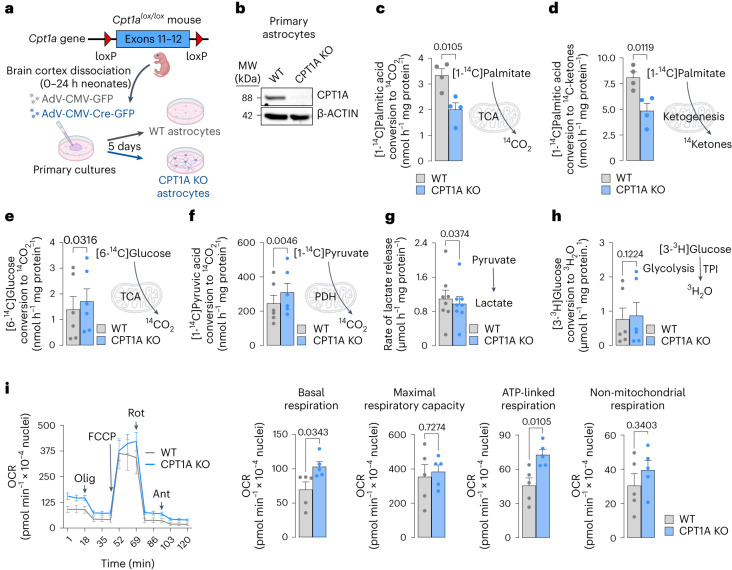
KO of *Cpt1a* in astrocytes inhibits fatty acid oxidation and metabolic rewiring enhancing mitochondrial oxygen consumption. **a**, Strategy used to obtain *Cpt1a* KO astrocytes in astrocytes in primary culture. Created with BioRender.com. **b**, Western blot against CPT1A protein in *Cpt1a* KO astrocytes in primary culture 5 days after AdV-CMV-Cre-GFP transduction; *n* = 3 biologically independent cell culture preparations; unpaired Student’s *t*-test, two-tailed. β-Actin was used as a loading control (Supplementary Fig. 2b). **c**, ^14^CO_2_ production from [1-^14^C]palmitic acid in WT and *Cpt1a* KO astrocytes in primary culture. Data are mean ± s.e.m. *P* value is indicated (*n* = 4 biologically independent samples; unpaired Student’s *t*-test, two-sided). **d**, ^14^Ketones production from [1-^14^C]palmitic acid in WT and *Cpt1a* KO astrocytes in primary culture. Data are mean ± s.e.m. *P* value is indicated (*n* = 4 biologically independent samples; unpaired Student’s *t*-test, two-sided). **e**–**h**, ^14^CO_2_ production from [6-^14^C]glucose (**e**) or [1-^14^C]pyruvic acid (**f**), rate of lactate released (**g**) and glycolytic flux as measured by the rate of [3-^3^H]glucose conversion into ^3^H_2_O (**h**), in WT and *Cpt1a* KO astrocytes in primary culture. TPI, triosephosphate isomerase. Data are mean ± s.e.m. *P* values are indicated; *n* = 6 (**e**), 6 (**f**), 8 (**g**) and 6 (**h**) biologically independent cell culture preparations; paired Student’s *t*-test, two-sided. **i**, OCR analysis and calculated parameters in WT and *Cpt1a* KO astrocytes in primary culture. Data are mean ± s.e.m. *P* values are indicated (*n* = 5 biologically independent cell culture preparations; unpaired Student’s *t*-test, two-sided) (Supplementary Fig. 2j,k). Source data

We next aimed to further characterize metabolically CPT1A KO astrocytes. To do so, astrocytes in primary culture from *Cpt1a*^*lox/lox*^ mice were transduced with adenoviruses expressing Cre recombinase under the potent cytomegalovirus (CMV) promoter (AdV-CMV-Cre-GFP) (Fig. 2a). *Cpt1a*^*lox/lox*^ astrocytes transduced with the AdV lacking Cre recombinase (AdV-CMV-GFP) were used as controls (WT). RT–qPCR analysis revealed *Cpt1a*, not *Cpt1b* or *Cpt1c*, mRNAs decrease in AdV-CMV-Cre-GFP transduced astrocytes (Supplementary Fig. 2a). As shown in Fig. 2b (Supplementary Fig. 2a), CPT1A protein was efficiently knocked out in AdV-CMV-Cre-GFP transduced astrocytes when compared with WT cells. CPT1B, CPT1C and CPT2 protein abundances were unaffected (Supplementary Fig. 2c). The release of 3-hydroxybutyrate from AdV-CMV-Cre-GFP transduced astrocytes was significantly reduced, suggesting impaired ketogenesis in CPT1A KO astrocytes (Supplementary Fig. 2d). We further assessed the rate of [1-^14^C]palmitic acid conversion to ^14^CO_2_ and to ^14^C-ketones, which were reduced by roughly 50% in CPT1A KO when compared with WT astrocytes (Fig. 2c,d and Supplementary Fig. 2e). Etomoxir, which inhibits both CPT1 (mitochondrial) and carnitine octanoyltransferase (peroxisomal)-mediated fatty acid uptake^21^, virtually abolished [U-^14^C]palmitic acid oxidation (Supplementary Fig. 2f). Thus, the cooperation of a cellular compartment, probably the peroxisome^22^, not depending on CPT1A, by converting long-chain into short-chain fatty acids is likely to contribute to the mitochondrial oxidation of fatty acids. Since astrocyte energy metabolism is thought to be largely sustained by glycolysis^3–5^, we next aimed to assess the impact of fatty acid oxidation on glucose metabolism. To do this, we first analysed the rate of [6-^14^C]glucose oxidation to ^14^CO_2_, a process that takes place in the tricarboxylic acid (TCA) cycle. As shown in Fig. 2e, [6-^14^C]glucose decarboxylation increased in CPT1A KO astrocytes. ^14^C_6_-Glucose via glycolysis labels ^14^C_3_-pyruvate, which decarboxylates exclusively at the TCA cycle depending on the rates of glycolysis, mitochondrial pyruvate import and pyruvate dehydrogenase (PDH) activity. To identify which of these steps accounts for the increased rate of [6-^14^C]glucose decarboxylation, we assessed [1-^14^C]pyruvate decarboxylation, which exclusively takes place at PDH, after mitochondrial pyruvate import. We found a significant increase in ^14^CO_2_ formation from [1-^14^C]pyruvate in CPT1 KO astrocytes (Fig. 2f), indicating enhanced PDH decarboxylation rate. This was validated by the assessment of ^293^Ser phosphorylation status of the PDH A1 (PDHA1) subunit, which was reduced (Supplementary Fig. 2g) indicating PDH activation^23^, an end that was confirmed by the PDH-specific activity (Supplementary Fig. 2h). This result, which explains the observed reduction in pyruvate concentration in the metabolomics analysis (Fig. 1e), indicates that, on *Cpt1a* loss, astrocytes undergo a metabolic rewiring consisting in enhanced pyruvate decarboxylation to acetyl-coenzyme A. Lactate is the main metabolic fate of glycolytically derived pyruvate in astrocytes^3–5^. We therefore assessed whether the enhanced mitochondrial pyruvate decarboxylation altered astrocytic-released lactate. As shown in Fig. 2g, lactate formation was reduced by a proportion (roughly 118 nmol h^−1^ mg protein^−1^) consistent with an increased pyruvate decarboxylation (roughly 65 nmol h^−1^ mg protein^−1^) (Fig. 2f) in CPT1A KO astrocytes, an effect that could not be accounted for by changes in the flux of glycolysis, as specifically measured by the rate of [3-^3^H]glucose conversion into ^3^H_2_O (Fig. 2h). These results indicate that, on *Cpt1a* KO, astrocytes rewire the metabolic fate of pyruvate to increase its mitochondrial oxidation without affecting glycolysis. Glycolysis and β-oxidation thus appear to be independently regulated pathways aimed to sustain different facets of astrocyte metabolism.

Given the contribution of fatty acid oxidation in sustaining astrocytic mitochondrial respiration (Supplementary Fig. 1b), we next analysed the OCR in CPT1A KO astrocytes. As shown in Fig. 2i, loss of CPT1A increased by roughly 1.5-fold the mitochondrial basal and ATP-linked respiration, without statistically significantly affecting maximal and non-mitochondrial respiration. These results apparently contrast with those showing decreased basal mitochondrial respiration by etomoxir (Supplementary Fig. 1b). However, etomoxir acutely inhibits CPT1, whereas the genetic deficiency of *Cpt1a* allows astrocytes to adapt to CPT1A loss. Thus, lack of CPT1A seems to reprogramme astrocytic metabolism to a conformation whereby the mitochondrial respiration is improved. Such an improvement is not the consequence of increased mitochondrial mass, according to protein abundance parameters (Supplementary Fig. 2i). Given that electron flux through mitochondrial CI is the most energetically efficient course to conserve mitochondrial energy, we analysed the proportion of mitochondrial respiration that is contributed by this complex. To do this, we inhibited mitochondrial respiration with CI-specific inhibitor rotenone, both in intact (Supplementary Fig. 2j) and in digitonin-permeabilized cells in the presence of CI-substrates and ADP (Supplementary Fig. 2k), which revealed that the contribution of CI to sustain mitochondrial respiration was significantly enhanced in CPT1A KO versus WT astrocytes. Altogether, these data indicate that mitochondrial oxidation of endogenous fatty acids in astrocytes preserves the mitochondrial respiratory chain conformation in an energetically less active mode.

**Fig. 3:**
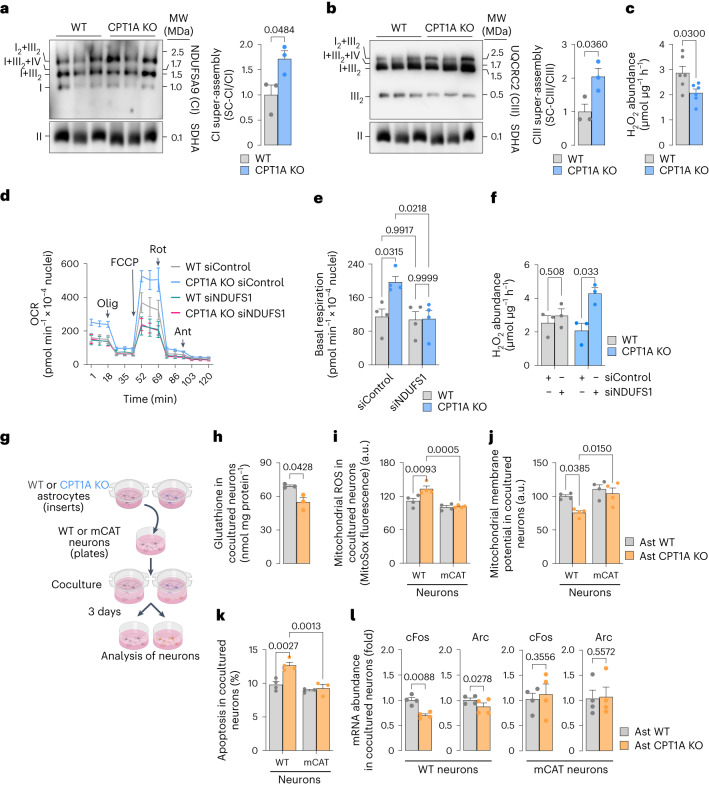
KO of *Cpt1a* in astrocytes induces mitochondrial SCs leading to increased respiration and decreased ROS affecting bioenergetics and function of cocultured neurons. **a**, Free CI and CI-containing SCs (SC-CI) in WT and *Cpt1a* KO primary astrocytes, analysed by BNGE followed by immunoblotting against CI subunit NDUFA9. Data are mean ± s.e.m. *P* values are indicated (*n* = 3 biologically independent cell culture preparations; unpaired Student’s *t*-test, two-sided). **b**, Free CIII and CIII-containing SCs (SC-CIII) in WT and *Cpt1a* KO primary astrocytes, analysed by BNGE followed by immunoblotting against CIII subunit UQCRC2. Data are mean ± s.e.m. *P* values are indicated (*n* = 3 biologically independent cell culture preparations; unpaired Student’s *t*-test, two-sided). **c**, H_2_O_2_ production in WT and CPT1A KO astrocytes in primary culture. Data are mean ± s.e.m. *P* values are indicated (*n* = 6 independent cell culture preparations; unpaired Student’s *t*-test, two-sided). **d**, OCR analysis in WT and *Cpt1a* KO astrocytes in primary culture, either transfected with scrambled (control) or NDUFS1 siRNAs. Data are mean ± s.e.m. *P* values are indicated (*n* = 4 biologically independent cell culture preparations) (Supplementary Fig. 3f). **e**, Basal respiration in WT and CPT1A KO astrocytes in primary culture, either transfected with scrambled (control) or NDUFS1 siRNAs. Data are mean ± s.e.m. *P* values are indicated (*n* = 4 biologically independent cell culture preparations; two-way ANOVA followed by Tukey). **f**, H_2_O_2_ production by WT and CPTA1A KO astrocytes in primary culture, either transfected with scrambled (control) or NDUFS1 siRNAs. Data are mean ± s.e.m. *P* values are indicated (*n* = 3 biologically independent cell culture preparations; multiple unpaired Student’s *t*-test). **g**, Strategy used to assess the effect of *Cpt1a* KO astrocytes on WT or mCAT neurons in primary culture. Created with BioRender.com. **h**–**l**, Glutathione concentration (**h**), mitochondrial ROS (**i**), ∆*ψ*_m_ (**j**), apoptosis (**k**) and c-Fos and Arc mRNA abundances (**l**) in WT or mCAT-expressing transgenic neurons after coculture with WT or *Cpt1a* KO astrocytes; *n* = 3 (**h**), 4 (**i**), 4 (**j**), 4 (**k**, WT), 4 (**k**, mitoCAT) and 4 (**l**) biologically independent cell culture preparations; paired Student’s *t*-test, two-sided for simple comparisons and two-way ANOVA followed by Tukey for multiple comparisons. Source data

To understand the molecular mechanism whereby fatty acid oxidation keeps less active mitochondrial respiration in astrocytes, we sought to investigate the super-assembly of the respiratory complexes, thought to regulate mitochondrial respiration^24–26^. To do so, mitochondria were isolated from CPT1A KO and WT astrocytes and their proteins subjected to blue native gel electrophoresis (BNGE) followed by western blotting against CI, CIII and CIV subunits. The analysis revealed that loss of CPT1A promoted a significant increase in mitochondrial supercomplexes (SC) formation (Fig. 3a,b and Supplementary Fig. 3a), probably explaining the observed increase in CI-sustained respiration (Supplementary Fig. 2j,k). The increased SC formation and CI-sustained respiration could not be ascribed to a putative enhancement in the protein abundances of the mitochondrial respiratory chain complexes (Supplementary Fig. 3b) nor in an enhancement in CI-specific activity (Supplementary Fig. 3c). The CI–III- and CIV-specific activities significantly increased in CPT1A KO astrocytes (Supplementary Fig. 3c), in coherence with enhanced mitochondrial respiration (Fig. 2i) and SC formation (Fig. 3a,b and Supplementary Fig. 3a). Even though it is the subject matter of debate^25,26^, besides the regulation of respiration, CI super-assembly in SC has been suggested to regulate reactive oxygen species (ROS) production in heart^27^ and brain cells including neurons and astrocytes^18^. Accordingly, we next investigated whether the observed super-assembly of the mitochondrial respiratory chain in CPT1A KO astrocytes had an impact on ROS abundance. As shown in Fig. 3c, hydrogen peroxide (H_2_O_2_) was significantly lower in CPT1A KO astrocytes. To establish a causal link between increased SC formation and decreased H_2_O_2_ generation in CPT1A KO astrocytes, we knocked down CI subunit NDUFS1 (Supplementary Fig. 3d), a strategy previously used to reduce CI levels^18^. This treatment caused disassembly of CI-containing SC in astrocytes (Supplementary Fig. 3e), prevented the increase in mitochondrial basal respiration (Fig. 3e) without statistically significantly affecting maximal, ATP-linked and non-mitochondrial respiration (Supplementary Fig. 3f), and increased H_2_O_2_ in the CPT1A KO astrocytes (Fig. 3f). Whereas higher-resolution structural work would be required to confirm this mechanism^25^, altogether, these data indicate that fatty acid oxidation keeps the astrocytic mitochondrial respiratory chain under less energetically favourable structural conformation that is able to sustain ROS generation.

**Fig. 4:**
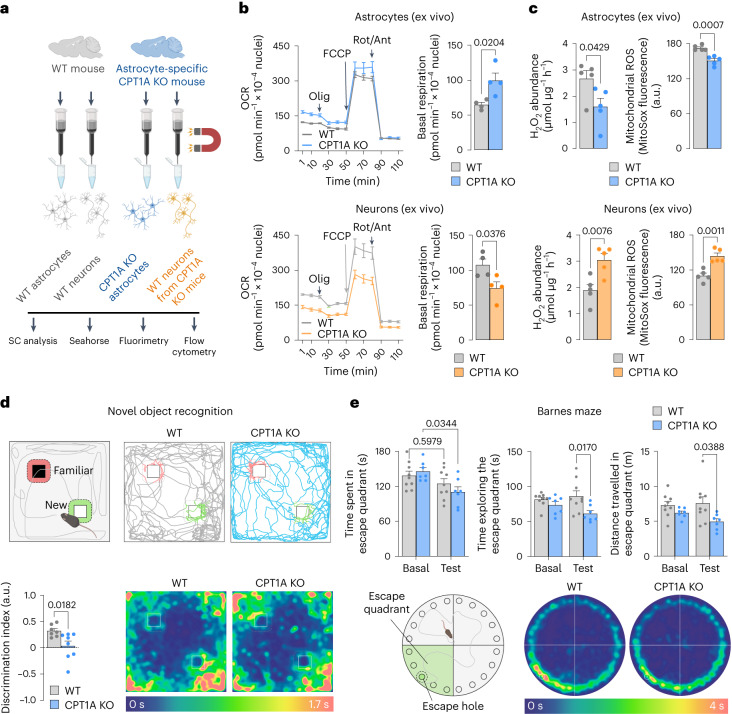
Astrocyte-specific *Cpt1a* KO mice enhance in astrocytes but decrease in neurons mitochondrial SCs and respiration causing cognitive impairment. **a**, Strategy used to immunomagnetically isolate astrocytes and neurons from WT or astrocyte-specific CPT1A KO adult mice. Created with BioRender.com. **b**, OCR analysis and calculated basal respiration in immunomagnetically isolated astrocytes (top) and neurons (bottom) from WT and astrocyte-specific *Cpt1a* KO mice. Data are mean ± s.e.m. *P* values are indicated (*n* = 4 mice per genotype; unpaired Student’s *t*-test, two-sided). **c**, H_2_O_2_ and mitochondrial ROS analyses in immunomagnetically isolated astrocytes (top) and neurons (bottom) from WT and astrocyte-specific *Cpt1a* KO mice. Data are mean ± s.e.m. *P* values are indicated (*n* = 5 mice per genotype; unpaired Student’s *t*-test, two-sided). **d**, Novel object recognition test in WT and astrocyte-specific *Cpt1a* KO mice. Representative paths and spatiotemporal quantitative heatmaps are shown. Data are mean ± s.e.m. *P* values are indicated (*n* = 9 (WT) or 7 (CPT1A KO) mice; unpaired Student’s *t*-test, two-sided). **e**, Barnes maze test in WT and astrocyte-specific *Cpt1a* KO mice 8 days after training. Spatiotemporal quantitative heatmaps are shown. Data are mean ± s.e.m. *P* values are indicated (*n* = 9 (WT) or 7 (CPT1A KO) mice; two-way ANOVA followed by Tukey). *P* values in the figure. Source data

Astrocyte ROS constitute redox signals that modulate brain metabolism to sustain mouse behaviour^28^. In addition, astrocytes are able to transform fatty acids into ketone bodies^10,29,30^ (Fig. 2d and Supplementary Fig. 2d) that, according to studies performed in *Drosophila*, may be shuttled to neurons for oxidative use^31,32^. Moreover, here we show that, as long as astrocytes oxidize fatty acids, pyruvate is converted to lactate, a metabolite that also may be shuttled to neurons^33,34^. Given that knocking out *Cpt1a* in astrocytes impaired ROS generation, which physiologically maintains neuronal integrity and mouse cognition^28^, we next investigated its impact on neuronal function and behaviour. Neurons cocultured with CPT1A KO astrocytes (Fig. 3g) underwent loss in antioxidant glutathione (Fig. 3h), in consonance with previous observations^28^, suggesting neuronal redox stress. In fact, these neurons showed increased mitochondrial ROS (Fig. 3i), mitochondrial membrane potential (∆*ψ*_m_) disruption (Fig. 3j) and apoptotic death (Fig. 3k). Neurons expressing a mitochondrial isoform of the antioxidant enzyme catalase (mitochondrial catalase (mCAT) neurons), which efficiently prevented the increased mitochondrial ROS caused by coincubation with CPT1A astrocytes (Fig. 3i), abolished ∆*ψ*_m_ loss and apoptosis (Fig. 3j,k). These bioenergetic alterations caused neuronal dysfunction, as judged by the reduced mRNA abundances of the neuronal functional markers^35,36^ *c-Fos* and *Arc* in a mitochondrial ROS-dependent manner (Fig. 3l). To ascertain the in vivo impact of our findings, we immunomagnetically isolated astrocytes from both WT and astrocyte-specific *Cpt1a* KO mice (Fig. 4a). Characterization of neural cell markers confirmed the purity of the ACSA^+^ (astrocytes) fraction in both genotypes (Supplementary Fig. 4a). Analysis of the mitochondrial respiratory chain SC in the mitochondrial fractions of these cells revealed that loss of CPT1A increased SC formation, an effect that was observed both in male and female mice (Supplementary Fig. 4b). Immunomagnetic isolation of neurons from astrocyte-specific *Cpt1a* KO mice (Fig. 4a), which resulted in an enriched fraction (Neuron^+^) according to the neural cell markers (Supplementary Fig. 4a), followed by SC analysis of their mitochondrial fractions, revealed CI disassembly from SC both in male and female mice (Supplementary Fig. 4c). These results indicate that loss of CPT1A in astrocytes causes mitochondrial dysfunction in neighbouring neurons. In good agreement with the data obtained in primary cultured astrocytes, astrocytes isolated from astrocyte-specific *Cpt1a* KO adult mice showed increased basal and ATP-linked respiration (Fig. 4b and Supplementary Fig. 4d), decreased H_2_O_2_ and mitochondrial ROS (Fig. 4c) with unchanged ∆ψ_m_ (Supplementary Fig. 4e) when compared with those isolated from WT mice. Neurons isolated from astrocyte-specific *Cpt1a* KO adult mice showed decreased basal and ATP-linked respiration (Fig. 4b and Supplementary Fig. 4d), increased H_2_O_2_ and mitochondrial ROS (Fig. 4c) with reduced ∆*ψ*_m_ (Supplementary Fig. 4e) when compared with those isolated from WT mice. Thus, neurons adjacent to CPT1A KO astrocytes develop adaptive changes that result in mitochondrial dysfunction and redox stress. To assess whether the observed effects on neurons have behavioural implications, mice were subjected to a battery of performance tests. The results revealed that astrocyte-specific *Cpt1a* KO mice did not develop a significant impairment in the open field performance (Supplementary Fig. 5a) or in the rotarod test (Supplementary Fig. 5b), indicating lack of anxiety and motor coordination. However, astrocyte-specific *Cpt1a* KO mice showed an impairment in the working memory, as judged by the observed decreased discrimination index in the novel object recognition test (Fig. 4d and Supplementary Fig. 5c), as well as an impairment in the long-term spatial memory according to the Barnes maze test (Fig. 4e and Supplementary Fig. 5d). Albeit through a different mechanism, a similar output takes place in neuron-specific *Cpt1c*-isoform genetic ablation^37^. Altogether, these results indicate that fatty acid oxidation in astrocytes is essential to maintain mitochondrial ROS formation, neuronal energy fitness and cognitive performance in mouse.

In conclusion, here we describe that mitochondrial oxidation of fatty acids in astrocytes exhibits signalling and higher-order cerebral advantages. This is mainly supported by the findings that astrocytic-specific genetic deletion of a key step in fatty acid use, CPT1A, impairs the physiological production of signalling mitochondrial ROS. In contrast to glucose catabolism that, via pyruvate oxidation, mainly conserves reducing equivalents as NADH(H^+^), fatty acid β-oxidation conserves both NADH(H^+^) and FADH_2_. Notably, in astrocytes, most fatty acid-derived acetyl-coenzyme A is converted into ketone bodies^10^ instead of the NADH(H^+^)-generating TCA cycle, thus strengthening the relative contribution of fatty acid-derived FADH_2_ to electron flux to CIII through electron transferring-ubiquinone oxidoreductase. Consistent with this, the relative mRNA expression of *Acadl* and *Mtpα*, responsible for long-chain fatty acid electron transfer to CIII (ref. ^16^), is high in astrocytes. By contrast, impairment of fatty acid oxidation by CPT1A loss adapts astrocyte metabolism towards increased pyruvate mitochondrial oxidation, CI assembly into SC and mitochondrial respiration. This mechanism is coherent with the recently proposed model^23^ that PDH activity converges with the functional organization of the mitochondrial respiratory chain to warrant optimal metabolic adaptations. Our data also confirm^18^ that mitochondrial respiratory chain is organized in astrocytes under a conformation in which CI is not fully assembled in SC, permitting fatty acids to contribute to mitochondrial respiration via electron transferring-ubiquinone oxidoreductase. Although this pathway is known to be less energetically efficient than CI-driven respiration, it allows a relatively high ROS generation^18^ with signalling purposes^28^. By granting preferential use of fatty acids, our results thus indicate that astrocytes prioritize the generation of mitochondrial ROS, essential for sustaining cognitive performance^28^, over a bioenergetic benefit (Supplementary Fig. 5e). Astrocytes are thought to largely meet their energy needs from glycolysis^3,7,38–41^, a pathway that here we show coexists with fatty acid oxidation. However, in light of our data, the contribution of these two pathways to bioenergetics and signalling functions are not analogous. Thus, although fatty acids are oxidized via the TCA cycle at a higher rate than glucose in astrocytes, fatty acids show higher respiration linked with ATP production, indicating more fuel is required for energy homeostasis. Glycolysis and fatty acid oxidation thus appear to be two pathways that each sustain essential facets of astrocyte metabolism and ROS signalling.

## Methods

### Cpt1a^*lox/lox*^ mice

All protocols were performed according to the European Union Directive 86/609/EEC and Recommendation 2007/526/EC, regarding the protection of animals used for experimental and other scientific purposes, enforced in Spanish legislation under the law 6/2013. Protocols were approved by the Bioethics Committee of the University of Salamanca or CIC bioGUNE (positron emission tomography and magnetic resonance spectroscopy) in accordance with the Spanish legislation (RD53/2013). *Cpt1a*^*lox/lox*^ mice were generated by introducing two loxP sites flanking a segment comprising exons 11 and 12 of *Cpt1a* gene via homologous recombination in embryonic stem cells under a C57BL/6J background^19^. Animals were bred at the Animal Experimentation Facility of the University of Salamanca in cages (maximum of five animals per cage) with a 12 h light and dark cycle (light from 08:00). The humidity was 45–65% and the temperature was 20–25 °C. Animals were fed ad libitum with a solid diet (20% proteins, 45% lipids and 35% carbohydrates, plus minerals and vitamins) and water.

### In vivo generation of astrocyte-specific *Cpt1a* KO mice

This was carried out using a validated AAV strategy^20^. Essentially, AAV particles of the PHP.eB capsid (serotype), known to efficiently transduce the central nervous system via intravenous injection^42^, expressing Cre recombinase driven by the astrocyte-specific short GFAP promoter (PHP.eB-AAV-gfaABC_1_D-Cre-GFP) were administered intravenously (50 µl aliquots of a phosphate-buffered saline (PBS) solution containing 0.001% Pluronic F-68, Sigma-Aldrich and 1 × 10^11^ viral genomes, VG) through the retro-orbital sinus to 2-month-old *Cpt1a*^*lox/lox*^ male mice under a brief sevoflurane anaesthesia (Sevorane, at 6% for initiation followed by roughly 3% for maintenance in air with supplements of O_2_ and NO_2_ 0.4 and 0.8 l min^−1^, respectively, using a gas distribution column, Hersill H-3, and a vaporizer, InterMed Penlons Sigma Delta). We used the retro-orbital sinus intravenous route because of the higher success rate observed when compared with the tail or temporal ones^43^. Siblings of WT mice received equivalent amounts of the same AAV particles that did not harbour Cre recombinase. Mice were used from 4 weeks after AAV injections.

### Primary cultures of astrocytes

Astrocytes in primary culture were obtained from the cortex of 0–24 h old *Cpt1a*^*lox/lox*^ mouse neonates^28^. Cell suspensions were seeded in 175 cm^2^ plastic flasks in low glucose (5.5 mM) Dulbecco’s Modified Eagle’s Medium (DMEM) supplemented with 10% fetal bovine serum and 4 mM glutamine, and incubated at 37 °C in a humidified 5% CO_2_-containing atmosphere. To detach non-astrocytic cells, after 7 days in vitro (DIV), the flasks were shaken at 150 r.p.m. overnight. The supernatant was discarded, and the attached, astrocyte-enriched cells were reseeded at 0.8–1 × 10^5^ cells per cm^2^ in the appropriate plates. Cells were used at 9 DIV. Individual primary cultures of mouse cortical neurons were prepared from E14.5 day-old mCAT^28^ or WT mice, seeded at 2.0 × 10^5^ cells per cm^2^ in six-well or Seahorse plates coated with poly-d-lysine (10 μg ml^−1^) and incubated in Neurobasal A supplemented with 2 mM glutamine, 5.5 mM glucose, 0.22 mM pyruvate and 2% antioxidant B27 supplement. Cells were incubated at 37 °C in a humidified 5% CO_2_-containing atmosphere. At 72 h after plating, the medium was replaced by 2% of the minus antioxidant (that is, lacking vitamin E, vitamin E acetate, superoxide dismutase, catalase and glutathione) B27 supplement. Neurons were used on day 6. To obtain astrocyte-neuronal cocultures, astrocytes at 8 DIV were reseeded on semipermeable polyester Transwell membrane inserts (4.5 cm^2^, 0.4 µm pore size; Corning) and allowed to attach for 24 h. After this time, astrocytes were transduced with the adenoviral particles and, after 4 days, astrocyte-containing inserts were placed over 3 DIV neurons and cocultured in Neurobasal A supplemented with 2 mM glutamine, 5.5 mM glucose, 0.22 mM pyruvate and 2% B27 minus antioxidant supplement for 3 days. Immunocytochemistry against a neuronal (β-Tubulin III: 1/300; T2200; Sigma), astrocytic (GFAP: 1/800; AB5541; Millipore), oligodendrocytes (O4; 1/300; from mouse hybridoma kindly donated by I. Fariñas’ laboratory) and microglial marker (CD45; 1/200; 553076; BD) was performed to determine the purity of the cultures, which was roughly 100% astrocytes (for astrocyte-enriched cultures) and 99.02% neurons, 0.43% astrocytes, 0.11% oligodendrocytes, 0.13% microglia and 0.31% other cells (for neuron-enriched cultures).

### Generation of *Cpt1a* KO astrocytes in primary culture

This was carried out by transducing 9 DIV primary astrocytes, obtained from *Cpt1a*^*lox/lox*^ mice, with adenoviral particles harbouring Cre recombinase driven by the ubiquitous citomegalovirus (CMV) promoter (AdV-CMV-Cre-GFP). Astrocytes from the same cultures transduced with equivalent amounts of the same AdV lacking Cre recombinase (Ad V-CMV-GFP) were used as WT astrocytes. Cells were used 5 days after transduction.

### Genotyping by PCR

For *Cpt1a*^*lox/lox*^ genotyping, a PCR with the following primers was performed 5′-CAGCTGCTCCACACCAAGGCT-3′ (forward) and 5′-TGCCCTTCTACTGTCACATGG-3′ (reverse), resulting in a 403 basepair (bp) band for *Cpt1*^*lox/lox*^ mice and 209 bp for WT^19^. PCR conditions were 30 s at 98 °C, 30 cycles of 5 s at 98 °C, 5 s at 60 °C, 10 s at 72 °C and a final extension of 2 min at 72 °C. Primers for genotyping the mCAT allele were 5′-CTCCCAAAGTCGCTCTGAGTTGTTATCA-3′, 5′-CGATTTGTGGTGTATGTAACTAATCTGTCTGG-3′ and 5′-GCAGTGAGAAGAGTACCACCATGAGTCC-3′, which yielded a 778-bp band for the WT allele and a 245-bp band for the mCAT allele. PCR conditions for mCAT genotyping were 5 min at 94 °C, 35 cycles of 30 s at 94 °C, 30 s at 65 °C, 3 min at 68 °C and 8 min at 68 °C. PCR products were resolved in 3% agarose gel using the 1 kilobase DNA ladder plus (Thermo Fisher Scientific).

### qPCR with reverse transcription

This was performed in total RNA samples, purified from primary cultures of astrocytes and neurons using the GenElute Mammalian Total RNA Miniprep Kit (Sigma), following the manufacturer’s protocol. Amplifications were performed in 100 ng of RNA, using Power SYBR Green RNA-to-CT 1-Step kit (Applied Biosystems). The primers were (forward and reverse, respectively) 5′-GGATGGCTATGGTCAAGGTC-3′ and 5′-GGCCTCACAGACTCCAGGTA-3′ for *Cpt1a*; 5′-TGCTCCATGGCAACTGCTAT-3′ and 5′-ACTCCCAGAGGTGCCCAAT-3′ for *Cpt1b*; 5′-CGCCCAGTATGAGAGGATGT-3′ and 5′-CCCTACACGGAAGAATCTGC-3′ for *Cpt1c*; 5′-GCAGTGGTCTTCGAGTGGAT-3′ and 5′-CAGCTGCCTTCAGACCATCA-3′ for *Acc1*; 5′-GAGTGGAAGCGGTCTCACAG-3′ and 5′-GCAAGCCTTCGTCCACATCC-3′ for *Acc2*; 5′-TCATTGCCAAGGCGGTTGAT-3′ and 5′-GCCATGGACTCAGTCACATAC-3′ for *Acadl*; 5′-CATGCGAATCCTCCAGGAAG-3′ and 5′-GCTACATCCACACCCACTTC-3′ for *Mtpα*; 5′-GGGAATGGTGAAGACCGTGT-3′ and 5′-CCGTTCCCTTCGGATTCTCC-3′ for *c-Fos*; 5′-CACTCTCCCGTGAAGCCATT-3′ and 5-TCCTCCTCAGCGTCCACATA-3′ for *Arc* and 5′-AGAGTCATGAGCTGCCTGAC-3′ and 5′-CAACGTCACACTTCATGATG-3′ for *β-actin*. The mRNA abundance of each transcript was normalized to that of *β-actin* obtained in the same sample. The resulting normalized values in astrocytes were expressed as the fold change versus the corresponding normalized values in neurons. When compared CPT1A KO with WT astrocytes, the resulting normalized values in CPT1A KO were expressed as the fold change versus the corresponding normalized values in WT astrocytes.

### Immunomagnetic purification of astrocytes and neurons from adult brain

Mouse adult brain (minus cerebellum and olfactory bulb) was dissociated using the adult mouse brain dissociation kit (Miltenyi Biotec). The tissue, once clean, was fragmented with a sterile scalpel in 2 ml per hemisphere of a disintegration solution (Earle’s Balanced Salt Solution, EBSS, 116 mM NaCl, 5.4 mM KCl, 1.5 mM MgSO_4_, NaHCO_3_ 26 mM, NaH_2_PO_4_·2H_2_O 1.01 mM, glucose 4 mM, phenol red 10 mg l^−1^, supplemented with albumin 14.4 μl ml^−1^ and DNase type I 26 μl ml^−1^, pH 7.2, trypsin 10.8 μl ml^−1^), and it was trypsinized at 37 °C in a thermostated bath for 5 min, shaking frequently to avoid decantation of the tissue. It was further mechanically disintegrated by trituration using a 5 ml serological pipette five times. Then, the suspension was returned to the thermostated bath for 10 min, shaking frequently. Trypsin activity was stopped by adding 10% fetal serum, before centrifuging the tissue at 700*g* for 5 min in a microfuge at 4 °C. Once the enzymatically disintegrated tissue had been decanted, the pellet was resuspended in a trypsin-free disintegration solution (EBSS + 13 μl ml^−1^ DNase + 20 μl ml^−1^ albumin) for mechanical trituration using a Pasteur pipette. Approximately five passages were performed per a volume of 4 ml and per hemisphere. The supernatant was centrifuged for 3 min at 700*g* and the number of cells in the pellet was counted. Once a homogeneous suspension of individualized adult neural cells was achieved, cell population separations were performed using MACS Technology using either the astrocyte-specific anti-ACSA-2 Microbead Kit or the neuron-specific Neuron Isolation Kit, according to the manufacturer’s protocol (MACS Technology). We confirmed the identity of the isolated fractions by western blotting against astrocytic (GFAP), neuronal (MAP2)-specific markers and the purity with microglial (Iba1) and oligodendroglial (OLIG2)-specific markers.

### Cell transfections

For NDUFS1 knockdown experiments, we used small interfering RNAs (siRNAs) against NDUFS1 (siNDUFS1; s105592; Life Technologies) and a siRNA control (siControl; 4390843; Life Technologies). Transfections with siRNAs were performed with Lipofectamine RNAiMAX reagent (Life Technologies) according to the manufacturer’s protocol using a siRNA final concentration of 9 nM. Cells were used after 3 days.

### Determination of metabolic fluxes

To assess fatty acid, glucose and pyruvate oxidative fluxes, we used radiometric approaches. To do this, astrocytes were seeded in 8 cm^2^ flasks hanging a microcentrifuge tube containing either 1 ml of benzethonium hydroxide (Sigma) (for ^14^CO_2_ equilibration) or 1 ml of H_2_O (for ^3^H_2_O equilibration). For brain slices, these were placed in 25-ml glass flasks harbouring a central well with a tube containing 0.8 ml of benzethonium hydroxide. All incubations were carried out in KRPG (NaCl 145 mM; Na_2_HPO_4_ 5.7 mM; KCl 4.86 mM; CaCl_2_ 0.54 mM; MgSO_4_ 1.22 mM; pH 7.35) containing 5 mM d-glucose at 37 °C in the air-thermostatized chamber of an orbital shaker. To ensure adequate oxygen supply for oxidative metabolism throughout the incubation period, the flasks’ atmospheres were gassed with carbogen (5% CO_2_/95% O_2_) before sealing them with rubber caps. To measure the carbon flux from fatty acids to CO_2_, cells (or brain slices) were incubated in KRPG (5 mM glucose) buffer with 0.25 µCi ml^−1^ of either [U-^14^C]- or [1-^14^C]palmitic acid (plus 10 µM palmitic acid)^44^, as indicated in the figures. To measure the carbon flux from glucose to CO_2_ through the TCA cycle, cells were incubated in KRPG (5 mM d-glucose) with 0.25 μCi ml^−1^ D-[6-^14^C]glucose^45^. To measure the carbon flux from pyruvate to CO_2_ through mitochondrial pyruvate uptake followed by PDH activity, cells were incubated in KRPG (5 mM d-glucose) with 0.25 μCi ml^−1^ [1-^14^C]pyruvate (plus 1 mM pyruvate). Incubations were terminated after 90 min by the addition of 0.2 ml 20% perchloric acid (Merck Millipore) and, after a further 60 min, the tube containing benzethonium hydroxide (with the trapped ^14^CO_2_) was used to determine the radioactivity using a liquid scintillation analyser (Tri-Carb 4810 TR, PerkinElmer). The glycolytic flux was measured by assaying the rate of ^3^H_2_O production from [3-^3^H]glucose using a similar strategy using 3 μCi ml^−1^ of d-[3-^3^H]glucose in KRPG buffer (5 mM d-glucose) for 120 min (ref. ^45^). After incubations were terminated with 0.2 ml 20% perchloric acid, the cells were further incubated for 96 h to allow for ^3^H_2_O equilibration with H_2_O present in the central microcentrifuge tube. The ^3^H_2_O was then measured by liquid scintillation counting (Tri-Carb 4810 TR, PerkinElmer). The specific radioactivity was used for the calculations. Under these experimental conditions, 75% of the produced ^14^CO_2_ and 28% of the produced ^3^H_2_O were recovered and were taken into account for the calculations^45^. To assess the conversion of fatty acids to ketones, astrocytes were seeded in 8 cm^2^ flasks with KRPG (5 mM glucose). The rate of ketone body formation was determined by adding 0.25 µCi ml^−1^ of [1-^14^C]palmitic acid bound to delipidated bovine serum albumin (BSA) (plus 10 µM palmitic acid) for 2 h. After incubations were terminated with 0.2 ml 20% (v/v) perchloric acid. Ketone bodies were extracted as a non-volatile, acid-soluble product^10^. To do this, 1 ml of the medium was taken and added to a delipidated 50 ml centrifuge tube (352070; FalconTM) containing 8 vols of a chloroform:methanol mixture (2:1, v/v) and 2 vols of KCl (0.1 M). After shaking, it was centrifuged for 5 min at 3,000*g*. The upper aqueous phase was taken and transferred to another tube containing 8 vols of chloroform:methanol mixture (2:1 v/v). After shaking and centrifuging under the same conditions, the upper aqueous phase was taken and transferred to a liquid scintillation vial for counting.

### Lactate determination

Lactate concentrations were measured in the culture medium spectrophotometrically^45^ by the determination of the increments in the absorbance of the samples at 340 nm in a mixture containing 1 mM NAD^+^, 8.25 U of lactate dehydrogenase in 0.25 M glycine, 0.5 M hydrazine and 1 mM ethylenediaminetetraacetic acid (EDTA) buffer, pH 9.5.

### β-Hydroxybutyrate determination

Astrocytes were incubated for 48 h in fresh medium, which was collected and snap frozen at −80 °C. β-Hydroxybutyrate was determined using a spectrophotometric-based detection kit (MAK134, Sigma) in 40 µl of samples following the manufacturer’s instructions.

### OCR assessment

OCRs of cell in primary culture or freshly immunomagnetically isolated were measured in real-time in an XFe24 Extracellular Flux Analyser (Seahorse Bioscience; Seahorse Wave Desktop software v.2.6.1.56). This equipment measures the extracellular medium O_2_ flux changes of cells seeded in XFe24-well plates. Regular cell medium was removed and washed twice with DMEM running medium (XF assay modified supplemented with 5 mM glucose, 2 mM l-glutamine, 1 mM sodium pyruvate, 5 mM HEPES, pH 7.4) and incubated at 37 °C without CO_2_ for 30 min to allow cells to pre-equilibrate with the assay medium. Oligomycin, FCCP (carbonyl cyanide-*p*-trifluoromethoxyphenylhydrazone) and a mixture of rotenone and antimycin, diluted in DMEM running medium, were loaded into port-A, port-B and port-C, respectively. Final concentrations in XFe24 cell culture microplates were 1 μM oligomycin, 2 μM FCCP, 1 μM rotenone and 2.5 μM antimycin. The sequence of measurements was as follows unless otherwise described. The basal level of OCR was measured three times, and then port-A was injected and mixed for 3 min, after OCR was measured three times for 3 min. Same protocol with port-B and port-C. OCR was measured after each injection to determine mitochondrial or non-mitochondrial contribution to OCR. All measurements were normalized to average three measurements of the basal (starting) level of cellular OCR of each well subtracting the non-mitochondrial OCR. Each sample was measured in 3–5 replicas. Experiments were repeated 3–5 times in biologically independent culture preparations. Non-mitochondrial OCR was determined by OCR after injection of antimycin plus rotenone together or separately. Maximal respiration was determined by maximum OCR rate after FCCP injection minus non-mitochondrial OCR. ATP production was determined by the last OCR measurement before oligomycin injection minus the minimum OCR measurement after oligomycin injection. When indicated, etomoxir (100 μM) was injected in port-A to determine fatty acids-dependent respiration, which was obtained by subtracting the minimum OCR value after etomoxir to that before etomoxir injection. To estimate CI-sustained mitochondrial respiration, OCR before rotenone injection was subtracted the minimum OCR measurement after rotenone injection, and then from this value the non-mitochondrial OCR was subtracted. We also determined CI-sustained respiration in permeabilized cells. To do so, XF DMEM buffer was switched to mannitol and sucrose (containing 70 mM sucrose, 220 mM mannitol, 10 mM KH_2_PO_4_, 5 mM MgCl_2_, 2 mM HEPES and 1 mM EGTA, pH 7.2) buffer and the basal OCR level monitored. To assess OCR in permeabilized cells, digitonin (25 µg ml^−1^), l-glutamine:malate (Gln:Mal; 4 mM:0.5 mM) and ADP (1 mM) were added to stimulate NAD^+^ reduction and respiration. Rotenone and antimycin were added sequentially to independently calculate CI-sustained respiration and non-mitochondrial OCR. CI-sustained respiration was calculated as the difference between OCR after digitonin/Gln/Mal/ADP and the OCR after antimycin (non-mitochondrial OCR).

### Specific activity of the mitochondrial respiratory complexes

Cells were collected and suspended in 10 mM phosphate buffer (KH_2_PO_4_; pH 7.0). After three cycles of freezing and thawing to ensure cellular disruption, the specific activities of CI, CI–III, CII–III, CIV and citrate synthase were determined. Rotenone-sensitive CI (NADH-ubiquinone oxidoreductase) activity^46^ was measured in KH_2_PO_4_ (25 mM, pH 7.2) in the presence of 10 mM MgCl_2_, 2.5 mg ml^−1^ BSA, 0.15 mM NADH and 1 mM KCN. Changes in absorbance at 340 nm (30 °C) (*ε* = 6.81 mM^−1^ cm^−1^) were recorded after the addition of 50 µM ubiquinone and 10 µM rotenone. CI–III (NADH-cytochrome *c* oxidoreductase) activity was determined in KH_2_PO_4_ (25 mM; pH 7.2) in the presence of 10 mM MgCl_2_, 50 mg ml^−1^ BSA, 300 mM KCN and 330 mM of oxidized cytochrome *c*. Changes in absorbance were recorded (550 nm; 30 °C) (*ε* = 19.2 mM^−1^ cm^−1^) after the addition of 10 mM NADH and 10 µM antimycin A plus 25 µM rotenone. CII–III (succinate-cytochrome *c* oxidoreductase) activity^47^ was determined in the presence of 100 mM phosphate buffer, plus 0.6 mM EDTA(K^+^), 2 mM KCN and 200 µM cytochrome *c*. Changes in absorbance were recorded (550 nm; 30 °C) (*ε* = 19.2 mM^−1^ cm^−1^) after the addition of 20 mM succinate and 10 µM antimycin A. For CIV (cytochrome *c* oxidase) activity, the first-rate constant, *k* (min^−1^ mg protein^−^^1^) of cytochrome *c* oxidation was determined^48^ in the presence of 10 mM phosphate buffer (KH_2_PO_4_; pH 7.0) and 50 µM reduced cytochrome *c*; absorbance was recorded every minute at 550 nm, 30 °C (*ε* = 19.2 mM^−1^ cm^−1^). Citrate synthase activity^49^ was measured in the presence of 93 mM of Tris-HCl, 0.1% (v/v) Triton X-100, 0.2 mM acetyl-CoA and 0.2 mM 5,5-dithio-*bis*-(2-nitrobenzoic acid) (DTNB); the reaction was started with 0.2 mM of oxaloacetate and the absorbance was recorded at 412 nm (30 °C) (*ε* = 13.6 mM^−1^ cm^−1^). Data were expressed as the ratio of the activities of each complex against the citrate synthase activity.

### PDH activity

PDH activity was determined by the reduction of NAD^+^ to NADH, coupled to the reduction of a reporter dye to yield a coloured reaction product with an increase in absorbance at 450 nm at room temperature, using the PDH Enzyme Activity Microplate Assay Kit (Abcam, catalogue no. ab109902) following the manufacturer’s instructions. Cell homogenates (300 μg of protein) were added to each well and the solubilized PDH enzyme was immunocaptured for 3 h. After washing twice with stabilizer, fresh assay solution was added and the absorbance of each well measured at 37 °C by a kinetic program at 450 nm for 30 min with a 60 s reading interval in a Varioskan Flash (Thermo Scientific). PDH activity (μOD × min^−1^) was expressed as the initial reaction rate determined from the slopes of the curves generated.

### Determination of glutathione concentrations

Cells were lysed with 1% (w/v) of sulfosalicylic acid, centrifuged at 13,000*g* for 5 min at 4 °C, and the supernatants were used for the determination of total glutathione (that is, reduced glutathione plus twice the concentration of oxidized glutathione), using oxidized glutathione (0–50 µM) as standard as described previously^50^. Total glutathione was measured in reaction buffer (0.1 mM NaHPO_4_, 1 mM EDTA, 0.3 mM DTNB, 0.4 mM NADPH, glutathione reductase 1 U ml^−1^, pH 7.5) by recording the increase in the absorbance after the reaction of reduced glutathione with DTNB for 2.5 min at 15 s intervals using a Varioskan Flash (Thermo Fisher) spectrophotometer (*λ* = 405 nm). Glutathione concentration (nmol mg^−1^ protein) was calculated from the slopes obtained in the samples, extrapolating them to those obtained in the standard.

### Flow cytometric detection of CPT1A

To assess the adenoviral particles-mediated Cre recombinase transduction efficiency in primary astrocytic cultures, cells were fixed, permeabilized using the Fix&Perm kit (Becton Dickinson Biosciences) and incubated with anti-CPT1A antibody (1/500) for 1 h at room temperature. Then, cells were incubated with the secondary Cy5-conjugated antibody for 30 min at room temperature and analysed in the FACScalibur flow cytometer (15 mW argon ion laser; CellQuest software, Becton Dickinson Biosciences) using FL1 and FL4 channels for GFP and CPT1A labelling, respectively.

### Flow cytometric analysis of apoptotic cell death

Cells were carefully detached from the plates using 1 mM EDTA (tetrasodium salt) in PBS (pH 7.4). APC-conjugated annexin-V and 7-amino-actinomycin D (7-AAD) (Becton Dickinson Biosciences) were used to determine quantitatively the percentage of apoptotic neurons by flow cytometry. Cells were stained with annexin-V-APC and 7-AAD in binding buffer (100 mM HEPES, 140 mM NaCl, 2.5 mM CaCl_2_), according to the manufacturer’s instructions, and 5 × 10^4^ cells were analysed, in three replicates per condition, on a FACScalibur flow cytometer (15 mW argon ion laser; CellQuest software, Becton Dickinson Biosciences), using FL4 and FL3 channels, respectively. Annexin^+^ and 7-AAD^−^ cells were considered apoptotic. The analyser threshold was adjusted on the flow cytometer channel to exclude most of the subcellular debris to reduce the background noise owing to the neurite disruption during neuronal detaching. Data were expressed as percentages.

### Metabolomics analysis

One hemisphere of 12-month-old WT or astrocyte-specific CPT1A KO mice (*n* = 6 for each condition) was snap frozen in liquid nitrogen and used for untargeted metabolomics analysis (Metabolon Incorporated). Ultrahigh performance liquid chromatography-tandem mass spectrometry analysis detected 751 metabolites. Raw data were extracted, peak-identified, quality-control processed, curated, normalized and log_2_ transformed by the Metabolon service. Peaks were quantified using the area under the curve. For studies spanning several days, a data normalization step was performed to correct variation resulting from instrument interday tuning differences. Essentially, each compound was corrected in run-day blocks by registering the medians to equal one (1.00) and normalizing each data point proportionately. Following imputation of missing values with the minimum observed value for each compound, Welch’s two-sample *t*-tests were used to identify compounds significantly different between experimental groups. Using these filters, we accepted a high estimate of the false discovery rate (*q* value ≤ 0.50), although other considerations were implemented to determine whether a result merited further scrutiny. Such evidence included (1) biological relevance given the genetic background context; (2) inclusion in a common pathway with a highly significant compound; (3) residing in a similar functional biochemical family with other significant compounds or (4) correlation with other experimental approaches. Graphs corresponding to statistical analysis were carried out with GraphPad v.8.0 and the online tool MetaboAnalyst v.5.0.

### Protein determinations

Protein samples were quantified by the BCA protein assay kit (Thermo) using BSA as a standard.

### Western blotting

Cells were lysed in RIPA buffer (1% sodium dodecylsulfate, 10 mM EDTA, 1% (vol/vol) Triton X-100, 150 mM NaCl and 10 mM Na_2_HPO_4_, pH 7.0), supplemented with protease inhibitor mixture (Sigma), 100 μM phenylmethylsulfonyl fluoride and phosphatase inhibitors (1 mM *O*-vanadate). Samples were boiled for 5 min. Aliquots of cell lysates (40 μg of protein) were subjected to SDS–PAGE on an 8 to 12% (vol/vol) acrylamide gel (MiniProtean; Bio-Rad) including PageRuler Prestained Protein Ladder (Thermo). The resolved proteins were transferred electrophoretically to nitrocellulose membranes (0.2 µm, Bio-Rad). Membranes were blocked with 5% (wt/vol) low-fat milk in TTBS (20 mM Tris, 150 mM NaCl and 0.1% (vol/vol) Tween 20, pH 7.5) for 1 h. Subsequent to blocking, membranes were immunoblotted with primary antibodies overnight at 4 °C. After incubation with horseradish peroxidase conjugated goat antirabbit IgG-HRP (1/10,000, Santa Cruz Biotechnologies), goat antimouse IgG-HRP (1/10,000, Bio-Rad), rabbit antigoat IgG-HRP (1/10,000, Abcam) or goat antirabbit IgG-HRP (1/3,000, Bio-Rad), membranes were immediately incubated with the enhanced chemiluminescence kit WesternBright ECL (Advansta), or Supersignal West Femto (Thermo) before exposure to Fuji Medical X-Ray film (Fujifilm) or using the Fusion FX transilluminator (Vilber GmbH). The protein abundances of all western blots per condition were measured by densitometry of the bands, in the linear phase of the exposure without reaching saturation, on the films or on the scanned autoradiograms using ImageJ v.1.48 software. At least three biologically independent replicates were always performed, although only one representative western blot is usually shown in the main figures.

### Primary antibodies for western blotting

Immunoblotting was performed with anti-CPT1A (1/1,000) (ab128568; Abcam), anti-CPT1B (1/1,000) (ab134988; Abcam), anti-CPT1C (1/1,000) (ab87498; Abcam), anti-CTP2 (1/1,000) (ab181114; Abcam), anti-heat-shock protein-60 (HSP60) (1/1,000) (ab46798; Abcam), anti-TOMM20 (1/1,000) (ab56783; Abcam), anti-NDUFS1 (1/500) (sc-50132; Santa Cruz Biotechnology), anti-UQCRC2 (1/1,000) (ab14745; Abcam), anti-GFAP (1/500) (G6171; Sigma), anti-NDUFB8 (1/1,000) (ab110242; Abcam), anti-NDUFA9 (1/1,000) (ab14713; Abcam), anti-SDHA (1/1,000) (ab14715; Abcam), anti-MTCO1 (1/1,000) (ab14705; Abcam), anti-COX IV (1/1,000) (ab16056; Abcam), anti-Iba1 (1/1,000) (019-19741; Wako), anti-MAP2 (1/1,000) (ab32454; Abcam), anti-OLIG2 (1/1,000) (ab109186; Abcam), anti-PDHA1 (1/1,000) (no. 3205; Cell Signaling), anti-phosphoSer^293^-PDHA1 (1/1,000) (no. 31866; Cell Signalling), anti-β-Tubulin III (1/300) (T2200; Sigma) and anti-β-actin (1/30,000) (A5441; Sigma).

### Mitochondrial isolation

To obtain the mitochondrial fraction, cell pellets were frozen at −80 °C and homogenized (10–12 strokes) in a glass-Teflon Potter–Elvehjem homogenizer in buffer A (83 mM sucrose and 10 mM MOPS; pH 7.2). The same volume of buffer B (250 mM sucrose and 30 mM MOPS) was added to the sample, and the homogenate was centrifuged (1,000*g*, 5 min) to remove unbroken cells and nuclei. Centrifugation of the supernatant was then performed (12,000*g*, 3 min) to obtain the mitochondrial fraction, which was washed in buffer C (320 mM sucrose; 1 mM EDTA and 10 mM Tris-HCl; pH 7.4)^18^. Mitochondria were suspended in buffer D (1 M 6-aminohexanoic acid and 50 mM Bis-Tris-HCl, pH 7.0).

### BNGE

For the assessment of complex I organization, digitonin-solubilized (4 g g^−1^) mitochondria (10–50 μg) were loaded in NativePAGE Novex 3–12% (vol/vol) gels (Life Technologies). After electrophoresis, in-gel NADH dehydrogenase activity was evaluated allowing the identification of individual complex I and complex I-containing SC bands due to the formation of purple precipitated at the location of complex I (ref. ^18^). Briefly, gels were incubated in 0.1 M of Tris-HCl buffer (pH 7.4), 1 mg ml^−1^ of nitro blue tetrazolium and 0.14 mM of NADH. Next, a direct electrotransfer was performed followed by immunoblotting against mitochondrial complex I antibody NDUFS1 and complex III antibody UQCRC2. Direct transfer of BNGE was performed after soaking the gels for 20 min (4 °C) in carbonate buffer (10 mM NaHCO_3_; 3 mM Na_2_CO_3_·10H_2_O; pH 9.5–10). Protein transfer to polyvinylidene fluoride membranes was carried out at 60 V for 90 min at 4 °C in carbonate buffer.

### Mitochondrial ROS

Mitochondrial ROS were determined with the fluorescent probe MitoSox (Life Technologies). Cultured cells or adult brain-cell suspensions were incubated with 2 μM of MitoSox for 30 min at 37 °C in a 5% CO_2_ atmosphere in Hank’s buffered saline solution (134.2 mM NaCl, 5.26 mM KCl, 0.43 mM KH_2_PO_4_, 4.09 mM NaHCO_3_, 0.33 mM Na_2_HPO_4_·2H_2_O, 5.44 mM glucose, 20 mM HEPES and 20 mM CaCl_2_·2H_2_O, pH 7.4). The cells were then washed with PBS (136 mM NaCl; 2.7 mM KCl; 7.8 mM Na_2_HPO_4_·2H_2_O; 1.7 mM KH_2_PO_4_; pH 7.4) and collected by trypsinization. MitoSox fluorescence intensity was assessed by flow cytometry (FACScalibur flow cytometer, BD Biosciences) and expressed in arbitrary units.

### H_2_O_2_ determination

For H_2_O_2_ assessments, AmplexRed (Life Technologies) was used. Cells were trypsinized and incubated in KRPG buffer (145 mM NaCl, 5.7 mM Na_2_HPO_4_, 4.86 mM KCl, 0.54 mM CaCl_2_, 1.22 mM MgSO_4_, 5.5 mM glucose, pH 7.35) in the presence of 9.45 μM AmplexRed containing 0.1 U ml^−1^ horseradish peroxidase. Luminescence was recorded for 2 h at 30 min intervals using a Varioskan Flash (Thermo Scientific) (excitation, 538 nm; emission, 604 nm). Slopes were used for calculations of the rates of H_2_O_2_ formation.

### Mitochondrial membrane potential

The mitochondrial membrane potential (Δ*ψ*_m_) was assessed with MitoProbe DilC_1_(5) (Life Technologies) (50 nM) by flow cytometry (FACScalibur flow cytometer, BD Biosciences) and expressed in arbitrary units. For this purpose, cell suspensions were incubated with the probe 30 min at 37 °C in PBS. Δ*ψ*_m_ are obtained after subtraction of the potential value determined in the presence of carbonyl cyanide-4-(trifluoromethoxy)phenylhydrazone (CCCP) (10 µM, 15 min) for each sample.

### Mouse perfusion, immunohistochemistry and imaging

Mice were anaesthetized by intraperitoneal injection of a mixture of xylazine hydrochloride (Rompun; Bayer) and ketamine hydrochloride:chlorbutol (Imalgene; Merial) (1:4, v/v) at 1 ml per kg body weight and then perfused intra-aortically with 0.9% NaCl to remove blood cells. After perfusion, brains were dissected out sagitally in two parts and postfixed with Somogyi (4% (wt/vol) paraformaldehyde and 10% (vol/vol) methanol, in 0.1 M phosphate buffer solution; pH 7.4) for 96 h at 4 °C. Brain blocks were rinsed three times with 0.1 M phosphate buffer solution and cryoprotected in 10, 20 and 30% (w/v) sucrose in phosphate buffer solution sequentially, until they sank. After immersion in optimal cutting temperature medium and freezing for at least 24 h, 30-μm-thick sagittal sections were obtained with a freezing-sliding cryostat (Leica). For immunohistochemistry, sections were incubated sequentially in (1) 5 mg ml^−1^ sodium borohydride in phosphate buffer solution for 30 min (to remove aldehyde autofluorescence); (2) three PBS washes of 10 min each; (3) 1/5,000 anti-GFP (ab290; Abcam) in 0.02% Triton X-100 (Sigma) and 5% goat serum (Jackson Immuno-Research) in 0.1 M phosphate buffer solution for 72 h at 4 °C; (4) three phosphate buffer solution washes of 10 min each; (5) fluorophore conjugated secondary antibody 1/500 Cy2 goat antirabbit (Jackson Immuno-Research) in phosphate buffer solution for 2 h at room temperature and (6) 0.5 μg ml^−1^ 4,6-diamidino-2-phenylindole in phosphate buffer solution for 10 min at room temperature. After being rinsed with phosphate buffer solution, sections were mounted with Fluoromount (Sigma) aqueous mounting medium and cover slips (Thermo Fisher). Sections were examined with epifluorescence and the appropriate filter sets under an Operetta CLS high-content imaging system (PerkinElmer). High-resolution images were acquired using an OperaPHX/OPRTCLS Air Objective ×20 hNA objective.

### Behavioural tests

Mice (1–9 months post-AAV injections, that is 3–12 months old) were left to acclimatize in the room for not less than 15 min in the same time slot of the day (14:00 to 20:00). Tracking was carried out once at a time and carefully cleaning the apparatus with 70% ethanol between trials to remove any odour cues. An ANY-box core was used, which contained a light grey base and an adjustable perpendicular stick holding a camera and an infrared photo-beam array to track the animal movement and to detect rearing behaviour, respectively. Mouse movements were tracked with the ANY-maze software and the AMi-maze interface to register all parameters described subsequently. For the open field test, a 40 × 40 × 35 cm (width, depth, height) black infrared transparent Perspex insert was used, and the arena was divided in three zones, namely the border (8 cm wide), centre (16% of total arena) and intermediate (the remaining area) zones. The test lasted for 10 min, and the distance travelled, the number of rearings and the time spent in each zone were measured. A rotarod test (Rotarod apparatus, Model 47600, Ugo Basile) was used to analyse motor balance and coordination. Mice were previously trained during three consecutive days, 2 days before the test. The rotarod conditions were a gradual acceleration from 4 to 25 r.p.m., reaching the final speed at 270 s. To analyse the short-time memory, we used the novel object recognition test (Stoelting) in a 40 × 40 × 35 cm (width, depth, height) core with black infrared transparent Perspex insert, also tracked with the ANY-maze software and the AMi-maze interface to register the track of the mice. Mice were accustomed to this environment for 10 min during two consecutive days, and the test was performed on the third day. Mice were left to explore two identical equidistant cubes for 5 min (the familiarization phase) and returned for 30 min into its cage. One cube was substituted for a similar size and colour sphere and mice were returned to the arena to explore the objects for other 5 min (the test phase). To score zone entries that consider the exploration of an object we consider the size of the object (3.8 × 3.8 cm) and the surrounding perimeter (6 × 6 cm). The ability to recognize the sphere as a novel object was determined as the discrimination index (DI), which was calculated as DI = (*T*_N_ − *T*_F_)/(*T*_N_ + *T*_F_), where *T*_N_ is the time spent exploring the new object (sphere) and *T*_F_ is the time spent exploring the familiar object (cube). For the Barnes maze test, we used an equipment consisting of a grey circular platform (120 cm in diameter elevated 90 cm above the floor). Along its perimeter there were 20 evenly spaced holes. The maze has one removable escape box that could be fitted under any of the holes and was filled with the animal bedding before each experiment. Black and white patterned pictures were used as spatial visual cues. All sessions were performed under a room lighting of 400 lux to increase the mouse aversion for the platform. The test consisted of three phases. First is the habituation phase, where the animals were left to explore the platform freely for 5 min 1 day before the training sessions. Afterwards, the animals underwent the training phase where they were allowed to locate the scape hole for a maximum of 5 min, for 3 days with four sessions per day. Finally, for the probe phase, mice were tested for spatial memory. In this session the escape box was removed, and the platform was virtually divided into four quadrants, each containing five holes. Mice were allowed to explore the maze for 5 min and the time spent in the quadrant that previously contained the escape box was quantified. The number of evaluated animals per test is specified in the figure legends (8 ≥ *n* ≤ 13).

### Statistical analysis

For simple comparisons, we used an unpaired two-tailed Student’s *t*-test and, eventually, multiple unpaired Student’s *t*-test, except for (1) Fig. 2e–h and (2) Fig. 3h,l, in which we used paired Student’s *t*-test (two-sided) because (1) each primary culture preparation and experiment were performed on a different day, and (2) in these experiments, neurons were obtained from the same preparation and then were exposed to two types of insert (those containing WT and those containing CPT1A KO astrocytes). For other multiple-values comparisons, we used two-way analysis of variance (ANOVA) followed by Tukey tests. All tests used are indicated in each figure legend. We carried out the statistical analysis using the GraphPad Prism v.8 software. The number of biologically independent culture preparations or animals used per experiment are indicated in the figure legends.

### Reporting summary

Further information on research design is available in the Nature Portfolio Reporting Summary linked to this article.

### Supplementary information

Supplementary Information Supplementary Figs. 1–5.

Reporting Summary

Supplementary Data 1 This file contains the source data of the Supplementary figures.

Supplementary Table 1 This file contains a table with the names, source, catalogue number and references of the antibodies used.

Supplementary Data 2 This file contains the unprocessed and uncropped western blots shown in the supplementary information of the paper.

Supplementary Data 3 This file contains representative plots of the flow cytometry analyses of the samples in the paper.

Supplementary Data 4 This file contains the tables with the statistics analyses of the Supplementary figures of the paper.

### Source data

Source Data Figs. 1–4 This file contains the source data of the main figures.

Source Data Figs. 1–3 This file contains the unprocessed and uncropped western blots shown in the main figures of the paper.

Source Data Figs. 1–4 This file contains the tables with the statistics analyses of the main figures of the paper.

## Data Availability

Source data are provided with this paper.
