# Supplementary material for: Fatty acid oxidation organizes mitochondrial supercomplexes to sustain astrocytic ROS and cognition
Source: Nat Metab. 2023 Jul 17;5(8):1290–302. doi: 10.1038/s42255-023-00835-6 (PMC10447235; doi:10.1038/s42255-023-00835-6)
Supplement: Supplementary file 1 — Supplementary Figs. 1–5. [file 42255_2023_835_MOESM1_ESM.pdf]

nature metabolism

Letter

https://doi.org/10.1038/s42255-023-00835-6

Fatty acid oxidation organizes mitochondrial supercomplexes to sustain astrocytic ROS and cognition

In the format provided by the authors and unedited

SUPPLEMENTARY FIGURES

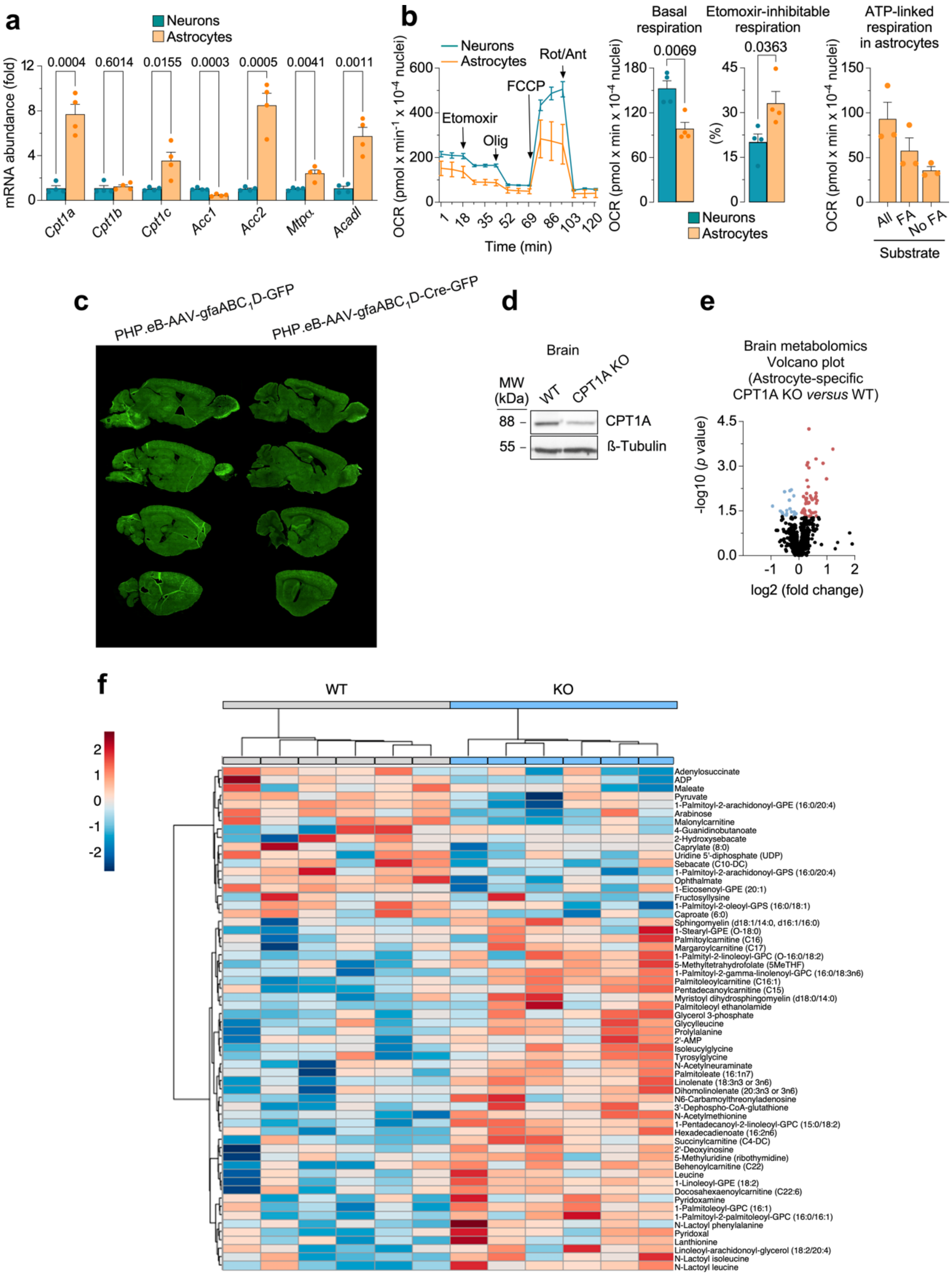

Supplementary Figure 1. CPT1A-dependent fatty acids utilization by astrocytes and impact on brain metabolomics.

(**a**) *Cpt1a*, *Cpt1b*, *Cpt1c*, *Acc1*, *Acc2*, *Mtp*α , and *Acadl* mRNA abundances in mouse astrocytes and neurons in primary culture. Data are mean ± S.E.M. *P* values are indicated (n=4 biologically independent cell culture preparations; Multiple unpaired Student's t test).

(**b**) OCR analysis and calculated basal respiration, etomoxir (100 µM)-inhibitable respiration and ATP-linked respiration in astrocytes and neurons in primary culture. Data are mean ± S.E.M. *P* values are indicated; n=4 (except for ATP-linked respiration, n=3) biologically independent cell culture preparations; Unpaired Student's t-test, two-sided.

(**c**) Brain distribution of GFP fluorescence 4 weeks after PHP.eB-AAV-gfaABC$_1$D-GFP and PHP.eB-AAV-gfaABC$_1$D-Cre-GFP intravenous retro-orbital injection in mice.

(**d**) Replica of the western blot shown in Fig. 1b showing the abundance of CPT1A in the brain of astrocyte-specific *Cpt1a* knockout *versus* WT mice.

(**e**) Volcano plot showing the abundance fold changes (X-axis) *versus* the statistical differences (*P* values, Y-axis) in the metabolomics analysis, colored dots representing metabolites statistically (P<0.05) different, in the brain of the astrocyte-specific *Cpt1a* knockout *versus* WT mice; n=6 mice per genotype, Welch's two-sample *t* tests with a false discovery rate of q value ≤ 0.50.

(**f**) Heatmap representation of the significantly altered metabolites observed in the brain of astrocyte-specific *Cpt1a* knockout when compared with WT mice analyzed by untargeted metabolomics.

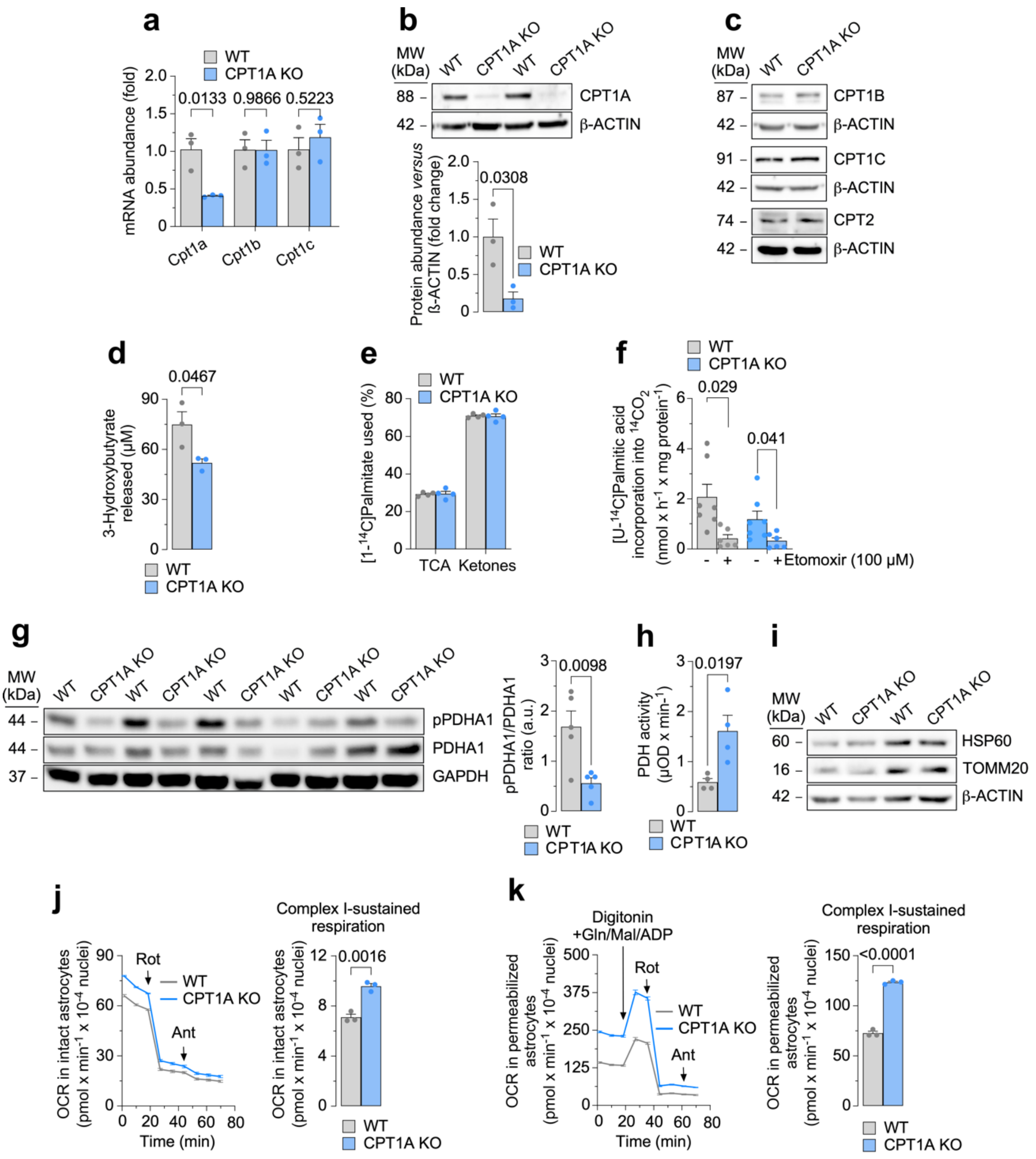

Supplementary Figure 2. *Cpt1a* knockout in astrocytes does not affect the other CPT isoforms or mitochondrial abundance but alters ketogenesis, PDH phosphorylation status and activity and CI-sustained respiration.

(a) *Cpt1a*, *Cpt1b* and *Cpt1c* mRNA abundances in astrocytes in primary culture from *Cpt1a*$^{lox/lox}$ mice 5 days after transduction with AdV-CMV-Cre-GFP (CPT1A KO) or AdV-CMV-GFP (WT). Data are mean ± S.E.M. *P* value is indicated (n=3 biologically independent cell culture preparations; multiple unpaired Student's t-test).

(**b**) Western blotting against CPT1A in *Cpt1a* knockout astrocytes in primary culture. ß-Actin was used as a loading control. Data are mean ± S.E.M. *P* values (KO *versus* WT)

are indicated (n=3 biologically independent cell culture preparations; Unpaired Student's t-test, two-tailed). Two samples are shown here, and one sample in Fig. 2b.

(**c**) Representative western blotting against CPT1B, CPT1C and CPT2 proteins in *Cpt1a* knockout astrocytes in primary culture. ß-Actin was used as a loading control.

(d) 3-Hydroxybutyrate concentration released to the medium of *Cpt1a* knockout astrocytes in primary culture in 48 hours. Data are mean ± S.E.M. *P* value is indicated (n=3 biologically independent cell culture preparations; Unpaired Student's t-test, two-tailed).

(e) Proportions of [1-^{14}C]palmitic acid incorporated into $^{14}CO_2$ or ^{14}C-ketones in *Cpt1a* knockout astrocytes in primary culture. This figure was generated from the data shown in Fig. 2c, d.

(**f**) Rate of $^{14}CO_2$ production from [U-^{14}C]palmitic acid in WT and *Cpt1a* knockout astrocytes in primary culture, either in the absence or in the presence of etomoxir. Data are mean ± S.E.M. *P* value is indicated; n=6 (with etomoxir) or 7 (without etomoxir) biologically independent samples; multiple unpaired Student's t-test.

(**g**) Western blotting against ^{293}Ser-phospho-pyruvate dehydrogenase A1 (pPDHA1) and PDHA1 in *Cpt1a* knockout astrocytes in primary culture. ß-Actin was used as a loading control. Data (pPDHA1/PDHA1 ratios) are mean ± S.E.M. *P* values (KO *versus* WT) are indicated (n=5 biologically independent cell culture preparations; unpaired Student's t-test, two-tailed).

(**h**) PDH activity in *Cpt1a* knockout astrocytes in primary culture. Data are mean ± S.E.M. *P* values (KO *versus* WT) are indicated (n=4 biologically independent cell culture preparations; unpaired Student's t-test, two-tailed).

(**i**) Western blots against HSP60 and TOMM20 in WT and *Cpt1a* knockout astrocytes in primary culture. Representative western blots of two independent experiments are shown.

(j) OCR in intact WT and *Cpt1a* knockout astrocytes in primary culture before and after incubation with rotenone and antimycin. The difference between OCR before and after rotenone incubation, minus OCR after antimycin, was used to calculate CI-sustained respiration. Data are mean ± S.E.M. *P* values (KO *versus* WT) are indicated (n=3 biologically independent cell culture preparations; unpaired Student's t-test, two-tailed).

(k) OCR in WT and *Cpt1a* knockout astrocytes in primary culture before and after incubation with digitonin (to permeabilize cells) plus glutamine (Gln), malate (Mal) and ADP. After this, cells were incubated with rotenone and antimycin. The difference between OCR before and after rotenone incubation, minus OCR after antimycin, was

used to calculate CI-sustained respiration. Data are mean ± S.E.M. *P* values (KO *versus* WT) are indicated (n=3 biologically independent cell culture preparations; unpaired Student's t-test, two-tailed).

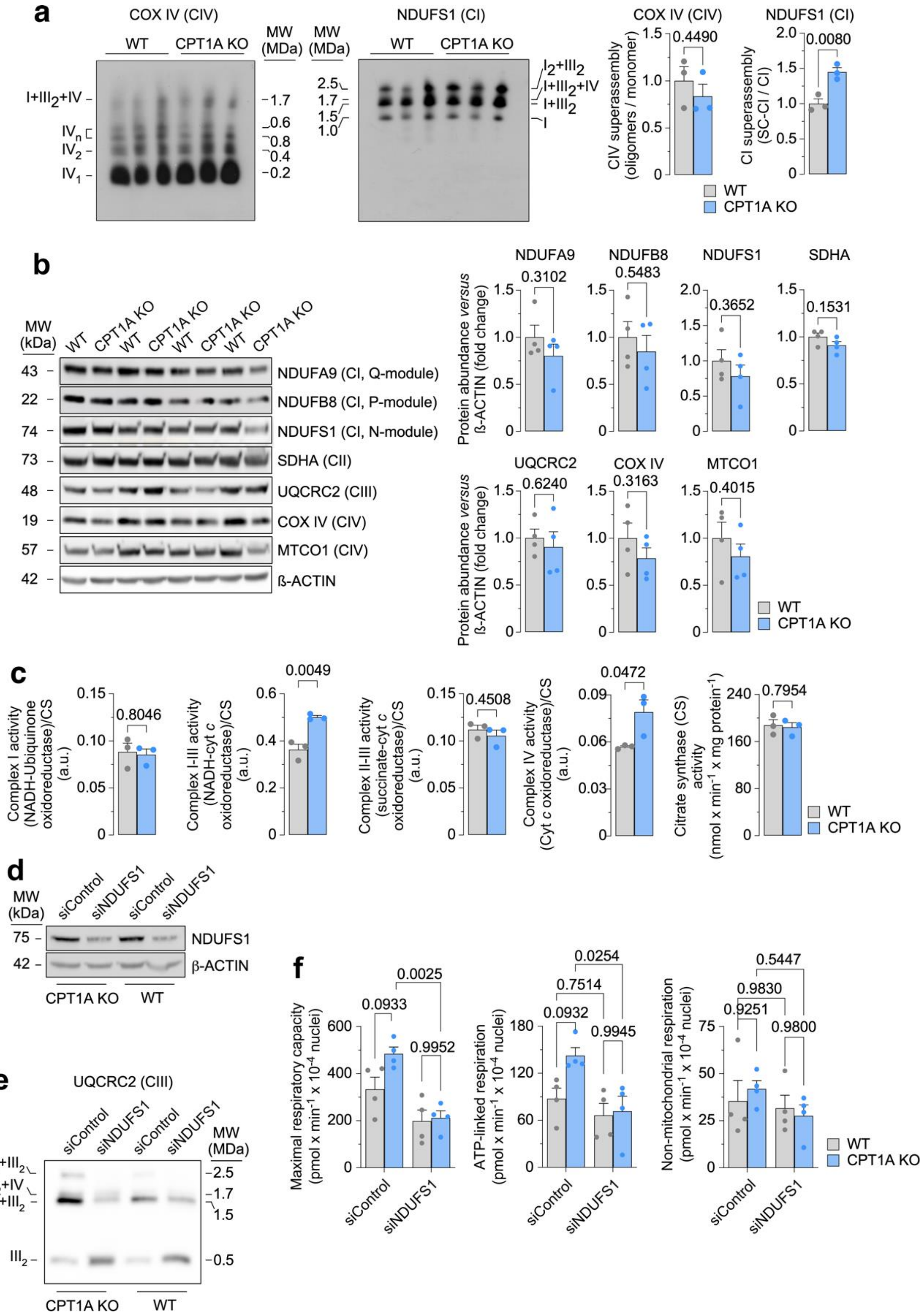

Supplementary Figure 3. Analysis of mitochondrial respiratory chain complexes activities and super-complexes formation in WT, *Cpt1a* knockout and NDUFS1 knockdown astrocytes.

(**a**) BNGE analysis of CIV monomer and oligomers, and CI superassembly in WT and *Cpt1a* knockout primary astrocytes. Data are mean ± S.E.M. *P* values are indicated (n=3 biologically independent cell culture preparations; unpaired Student's t-test, two-sided).
(b) Western blot analysis of the protein expression of complexes I, II, III, and IV subunits in WT and *Cpt1a* knockout astrocytes in primary culture. Data are mean ± S.E.M. *P* values are indicated (n=4 biologically independent cell culture preparations; unpaired Student's t-test, two-sided).
(c) Mitochondrial respiratory chain complex I, I-III, II-III, IV and citrate synthase activities in WT and *Cpt1a* knockout astrocytes in primary culture. Data are mean ± S.E.M. *P* values are indicated (n=3 biologically independent cell culture preparations; unpaired Student's t-test, two-sided).
(**d**) Representative (n=1) western blot against NDUFS1 in WT and *Cpt1a* knockout astrocytes in primary culture, either transfected with scrambled (control) or NDUFS1 siRNAs (siNDFUS1).
(**e**) Representative (n=1) BNGE showing free CIII and CIII-containing supercomplexes I-III (SC-CIII) in WT and *Cpt1a* knockout primary astrocytes, either in transfected with scrambled (control) or NDUFS1 siRNAs (siNDFUS1).
(**f**) OCR calculated parameters in WT and *Cpt1a* knockout astrocytes transfected with either siControl or siNDUFS4 . Data are mean ± S.E.M. *P* values are indicated (n=4 mice per genotype; two-way ANOVA followed by Tukey).

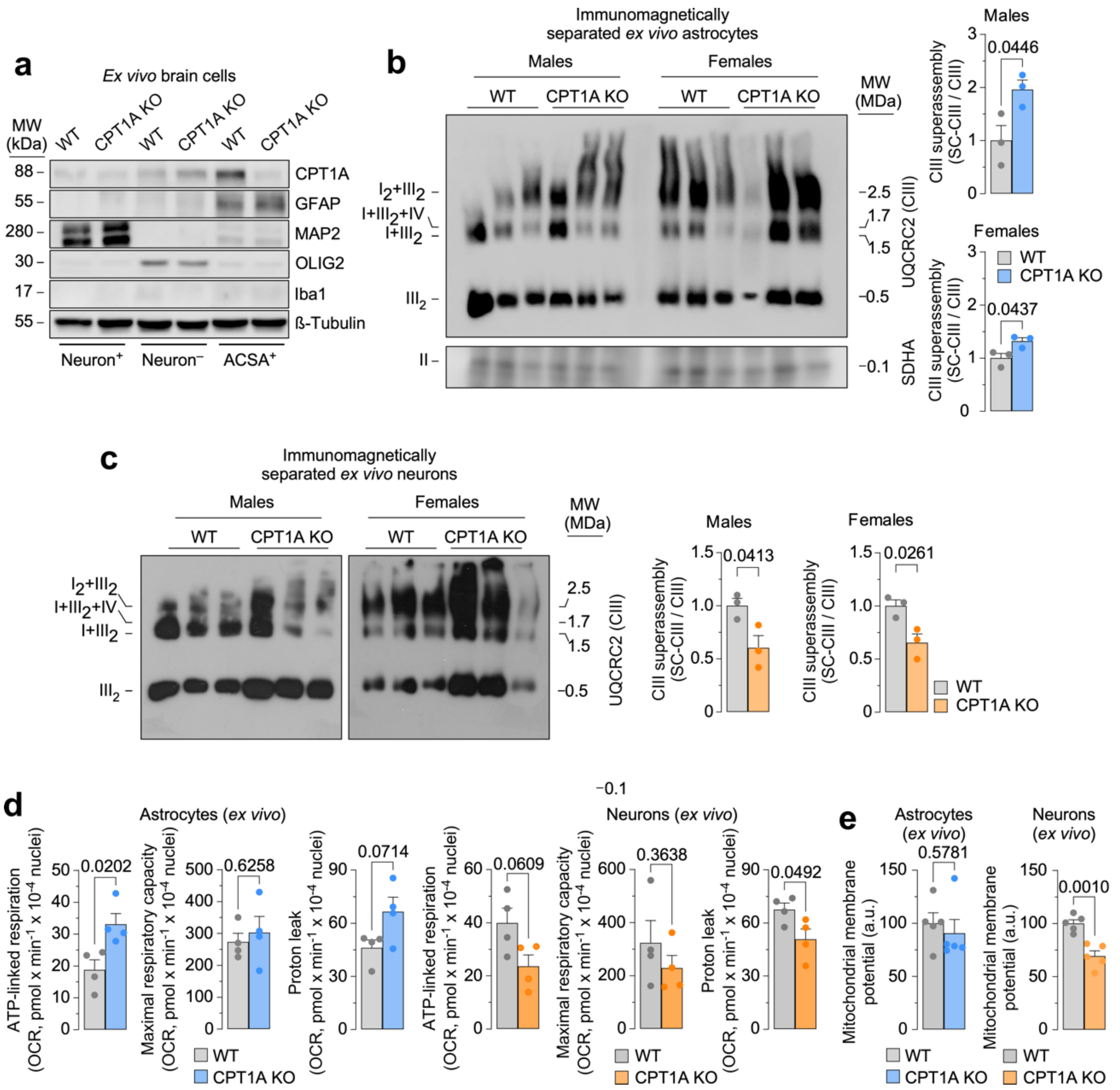

Supplementary Figure 4. *Ex vivo* astrocyte and neuron analysis in *Cpt1a* knockout mice.

(**a**) Representative western blotting against CPT1A, GFAP, MAP2, OLIG2, and Iba1 proteins in ACSA$^+$ (astrocytes), Neuron$^+$ (neurons), and Neuron$^-$ (glia) cells, immunomagnetically isolated from astrocyte-specific *Cpt1a* knockout mouse brain. ß-Tubulin was used as a loading control.

(b) Mitochondrial supercomplexes in immunomagnetically isolated astrocytes from WT and astrocyte-specific *Cpt1a* knockout male and female mice. Data are mean ± S.E.M. *P* values are indicated (n=3 mice per genotype; unpaired Student's t-test, two-sided).

(c) Mitochondrial supercomplexes in immunomagnetically isolated neurons from WT and astrocyte-specific *Cpt1a* knockout male and female mice. Data are mean ± S.E.M. *P* values are indicated (n=3 mice per genotype and sex; unpaired Student's t-test, two-sided).

(**d**) OCR calculated parameters in immunomagnetically isolated astrocytes and neurons from WT and astrocyte-specific *Cpt1a* knockout mice. Data are mean ± S.E.M. *P* values are indicated (n=4 mice per genotype; Student's t-test, one-sided).

(**e**) Mitochondrial membrane potential in immunomagnetically isolated astrocytes and neurons from WT and astrocyte-specific *Cpt1a* knockout mice. Data are mean ± S.E.M. *P* values are indicated (n=5 mice per genotype; Student's t-test, one-sided).

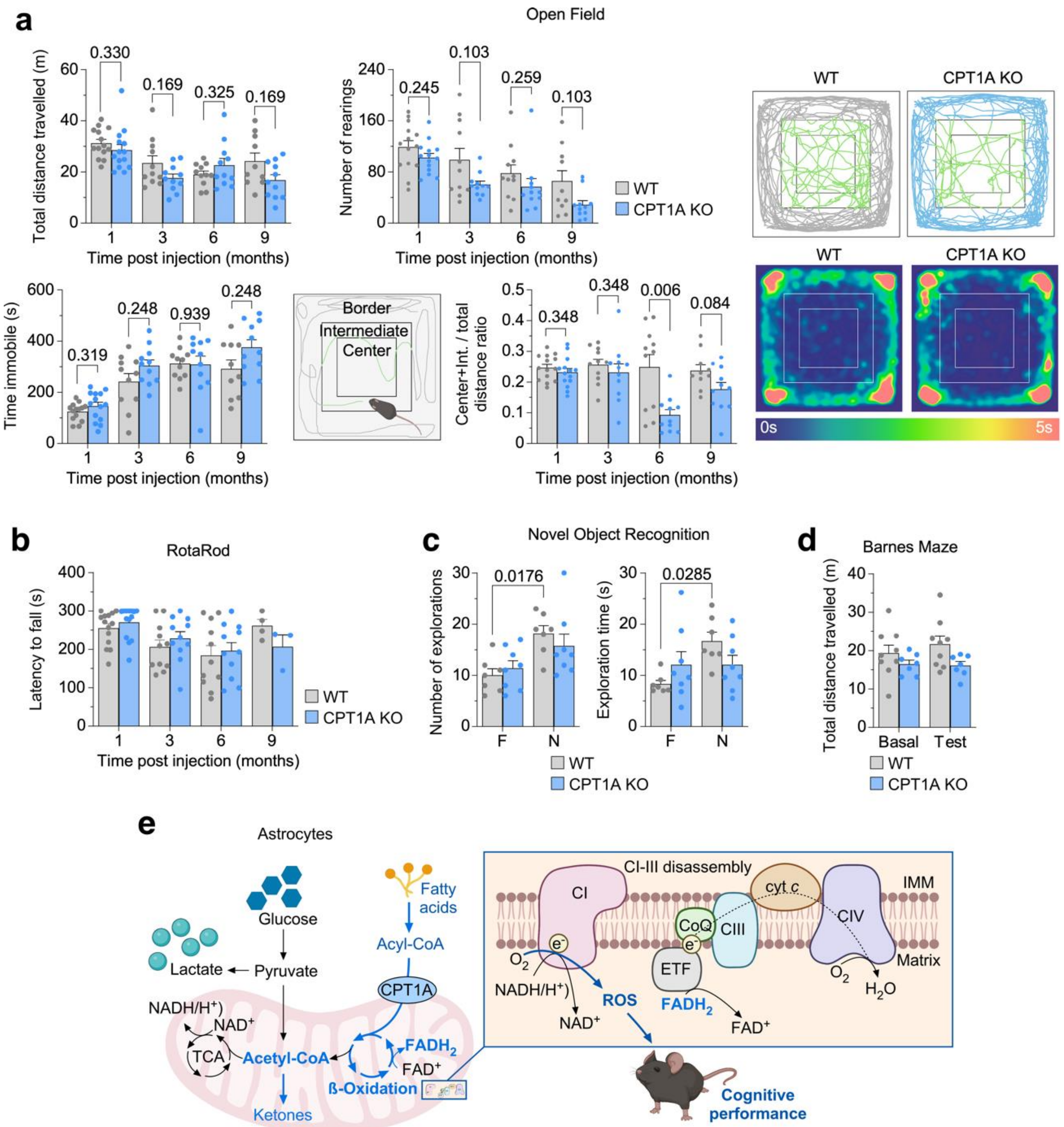

Supplementary Figure 5. *In vivo* behavioral analysis in *Cpt1a* knockout mice.

(**a**) Open field test in WT and astrocyte-specific *Cpt1a* knockout mice at different ages. Data are mean ± S.E.M. *P* values are indicated; n=14 (1 month), 11 (3 months), 11 (6 months), 11 (9 months, WT) or 10 (9 months, CPT1A KO) mice per genotype; multiple unpaired Student's t-test.

(**b**) Rotarod test in WT and astrocyte-specific *Cpt1a* knockout mice at different ages. Representative paths are shown. Data are mean ± S.E.M. *P* values are indicated; n=14 (1 month), 11 (3 months), 11 (6 months), 4 (9 months, WT) or 3 (9 months, CPT1A KO) mice per genotype.

(**c**) Number of explorations and exploration time in the novel object recognition test in WT and astrocyte-specific *Cpt1a* knockout mice. Data are mean ± S.E.M. P values are

indicated; n=7 (WT) or 8 (CPT1A KO) mice per genotype; two-way ANOVA followed by Tukey.

(**d**) Total distance traveled in the Barnes maze test in WT and astrocyte-specific *Cpt1a* knockout mice 8 days after training. Data are mean ± S.E.M. *P* values are indicated; n=9 (WT) or 7 (CPT1A KO) mice per genotype.

(**e**) Graphical summary describing the main message of this work. We show that in astrocytes, fatty acids-derived FAD-linked electrons ($FADH_2$), through the electron transferring flavoprotein (ETF), access ubiquinone (CoQ) and CIII bypassing CI, which consequently disassembles from CIII to form a less energetically efficient mitochondrial respiratory chain arrangement that increases ROS generation. Fatty acid oxidation to NAD-linked electron flux to mitochondrial CI is negligible since a large proportion of fatty acids-derived acetyl-coenzyme A (acetyl-CoA) is converted into ketone bodies in astrocytes. Thus, astrocytes prioritize the generation of mitochondrial ROS, known to be essential in sustaining cognitive performance, over a bioenergetic advantage. IMM, inner mitochondrial membrane.
