# Supplementary material for: Fatty acid oxidation organizes mitochondrial supercomplexes to sustain astrocytic ROS and cognition
Source: Nat Metab. 2023 Jul 17;5(8):1290–302. doi: 10.1038/s42255-023-00835-6 (PMC10447235; doi:10.1038/s42255-023-00835-6)
Supplement: Supplementary file 2 — Reporting Summary [file 42255_2023_835_MOESM2_ESM.pdf]

nature portfolio

Corresponding author(s): Juan P. Bolaños
Marina Garcia-Macia
Angeles Almeida

Last updated by author(s): 28/5/23

Reporting Summary

Nature Portfolio wishes to improve the reproducibility of the work that we publish. This form provides structure for consistency and transparency in reporting. For further information on Nature Portfolio policies, see our Editorial Policies and the Editorial Policy Checklist.

Statistics

For all statistical analyses, confirm that the following items are present in the figure legend, table legend, main text, or Methods section.

n/a	Confirmed	
☐	☒	The exact sample size (n) for each experimental group/condition, given as a discrete number and unit of measurement
☐	☒	A statement on whether measurements were taken from distinct samples or whether the same sample was measured repeatedly
☐	☒	The statistical test(s) used AND whether they are one- or two-sided<br>*Only common tests should be described solely by name; describe more complex techniques in the Methods section.*
☐	☒	A description of all covariates tested
☒	☐	A description of any assumptions or corrections, such as tests of normality and adjustment for multiple comparisons
☐	☒	A full description of the statistical parameters including central tendency (e.g. means) or other basic estimates (e.g. regression coefficient) AND variation (e.g. standard deviation) or associated estimates of uncertainty (e.g. confidence intervals)
☐	☒	For null hypothesis testing, the test statistic (e.g. F, t, r) with confidence intervals, effect sizes, degrees of freedom and P value noted<br>*Give P values as exact values whenever suitable.*
☒	☐	For Bayesian analysis, information on the choice of priors and Markov chain Monte Carlo settings
☒	☐	For hierarchical and complex designs, identification of the appropriate level for tests and full reporting of outcomes
☒	☐	Estimates of effect sizes (e.g. Cohen's d, Pearson's r), indicating how they were calculated

Our web collection on statistics for biologists contains articles on many of the points above.

Software and code

Policy information about availability of computer code

Data collection	CellQuest Pro v5, Tecnai™ Spirit TEM, QuantaSmart for TriCarb 5.1 (Perkin Elmer), AMi-maze® interface and ANY-maze® 5.33 software, Seahorse Wave Desktop Software 2.6.1.56, BioRad CFX Maestro 1.1, Harmony 4.9, Fluoview FV10-ASW, Vilber® Fusion FX6 Edge
Data analysis	Adobe Photoshop 12, ImageJ 1.48V, Paint-A-Gate ™ PRO, GraphPad Prism v8.0, IBM SPSS 23.0, FlowJo v10, Microsoft Excel (Microsoft 365) , Fluoview FV10-ASW, Seahorse Wave Desktop Software 2.6.1.56, MetaboAnalyst 5.0

For manuscripts utilizing custom algorithms or software that are central to the research but not yet described in published literature, software must be made available to editors and reviewers. We strongly encourage code deposition in a community repository (e.g. GitHub). See the Nature Portfolio guidelines for submitting code & software for further information.

Data

Policy information about availability of data

All manuscripts must include a data availability statement. This statement should provide the following information, where applicable:

- Accession codes, unique identifiers, or web links for publicly available datasets
- A description of any restrictions on data availability
- For clinical datasets or third party data, please ensure that the statement adheres to our policy

Source data for each figure are provided with this paper: the raw data and original immunoblots that support all the figures and findings of this study are available in the Source Data file and Supplementary Information.

Human research participants

Policy information about studies involving human research participants and Sex and Gender in Research.

Reporting on sex and gender	N/A
Population characteristics	N/A
Recruitment	N/A
Ethics oversight	N/A

Note that full information on the approval of the study protocol must also be provided in the manuscript.

Field-specific reporting

Please select the one below that is the best fit for your research. If you are not sure, read the appropriate sections before making your selection.

☒ Life sciences ☐ Behavioural & social sciences ☐ Ecological, evolutionary & environmental sciences

For a reference copy of the document with all sections, see nature.com/documents/nr-reporting-summary-flat.pdf

Life sciences study design

All studies must disclose on these points even when the disclosure is negative.

Sample size	We estimated the sample sizes for each type of experiment according to the ARRIVE guidelines in order to obtain a minimum power of 0.8 (80%) with the fewest possible animals (Krzywinski & Altman, Power and sample sizes, Nat Methods 2013, 10:1139, doi.org/10.1038/nmeth.2738; Button et al., Power failure: why small sample size undermines the reliability of neuroscience, Nat Rev Neurosci 2013, 14:365, doi.org/10.1038/nrn3475). We optimized the experimental protocols by performing pilot experiments in order to maximize the differences between conditions. In addition, we estimated the sample sizes by the previous experience of the group in in vitro molecular experiments and in vivo experiments and/or on previously published similiar experiments.<br>Exact information on the sample numbers being analyzed can be found in Figure legends and in Supplementary Information.<br>The majority of biochemical assays were repeated at least three times in order to derive statistical information such as error bars, p values and significance.
Data exclusions	No data were excluded from analyses.
Replication	In vitro: experiments were done from 3 to 5 times with independent biological samples and the necessary technical replicates for each technique (tipically, 4-6 replicas), in order to reproduce the results found. In vivo: the sample size of the behavioral studies was higher than in the in vitro experiments given the variability in the parameters measured in order to confirm a reliable result. This information has been added in the Statistical section of the manuscript. All attempts at replication were successfull. In vivo experiments were performed in 3-14 mice (usually 10-11) per condition.
Randomization	For all mouse experiments, animals were chosen based on genotypes. Aged-matched wild-type and mutant littermates were compared to minimize variance in age, genetic background and environment. Then a general method of randomization to assign experimental groups was not performed because all experiments were conducted with appropiate positive and negative controls, therefore it was not applicable.<br>For in vitro studies, randomization is not applicable as cells with different treatments or genetic knockdown cannot be randomized.
Blinding	Blinding was not considered to be necessary in biochemical, blotting and imaging experiments because they were analyzed in exactly the same manner.<br>For in vivo analysis, all experiments were done by experienced researchers blind for the experimental conditions.

Reporting for specific materials, systems and methods

We require information from authors about some types of materials, experimental systems and methods used in many studies. Here, indicate whether each material, system or method listed is relevant to your study. If you are not sure if a list item applies to your research, read the appropriate section before selecting a response.

Materials & experimental systems

n/a	Involved in the study	
☐	☒	Antibodies
☒	☐	Eukaryotic cell lines
☒	☐	Palaeontology and archaeology
☐	☒	Animals and other organisms
☒	☐	Clinical data
☒	☐	Dual use research of concern

Methods

n/a	Involved in the study	
☒	☐	ChIP-seq
☐	☒	Flow cytometry
☒	☐	MRI-based neuroimaging

Antibodies

Antibodies used

Primary antibodies:

CPT1a (clone 8F6AE9, ab128568; Abcam, 1/1000), CPT1b (ab134988; Abcam, 1/1000), CPT1c (b87498; Abcam, 1/1000), CPT2 (clone EPR13626, ab181114; Abcam, 1/1000), GFAP (clone 2e8, G6171; Sigma ,1/500), HSP60 (ab46798; Abcam, 1/1000), NDUFS1 (clone E-20, sc-50132; Santa Cruz Biotechnology 1/500), TOMM20 (clone 4F3, ab56783; Abcam, 1/1000), UQCRC2 (clone 13G12AF12BB11, ab14745; Abcam, 1/1000), B-Actin (clone AC-15, A5441; Sigma ,1/30000), B-Tubulin III (ab18207; Abcam ,1/500), NDUFB8 (clone 20E9DH10C12, ab110242; Abcam; 1/1000), NDUFA9 (clone 20C11B11B11, ab14713; Abcam, 1/1000), SDHA (clone 2E3GC12FB2AE2, ab14715; Abcam, 1/1000), MTCO1 (clone 1D6E1A8, ab14705; Abcam, 1/1000), COX IV (ab16056; Abcam, 1/1000), IBA1 (019-19741; Wako, 1/1000), MAP2 (ab32454; Abcam, 1/1000), OLIG2 (clone EPR2673, ab109186; Abcam, 1/1000), PDH (clone C54G1, #3205; Cell Signaling, 1/1000), pSer293-PDH (#31866; Cell Signaling, 1/1000), GFP (ab290; Abcam, 1/5000), GFAP (G9269; Sigma, 1/500).

Secondary antibodies:

Goat anti-mouse IgG-HRP (170-6516; Bio-Rad, 1/10000), rabbit anti-goat IgG-HRP (sc-2768, Santa Cruz, 1/10000), goat anti-rabbit IgG-HRP (170-6515; Bio-Rad, 1/10000), goat anti-rabbit-Cy2 (111-225-144, Jackson Immunoresearch, 1/500), goat anti-mouse-Cy5 (115-175-003, Jackson Immunoresearch, 1/500).

Validation

Antibodies used in this study were validated by the manufacturer who provided references on their websites using the catalog number provided and/or proven to work in the following papers (references belong):
- CPT1a (ab128568; Abcam, 1/1000) was already employed in Horie T et al. 2012; Lounis MA et al. 2017; Wang L et al. 2021; among others.
- CPT1b (ab134988; Abcam, 1/1000) was already employed in Eleftheriadis T et al. 2016; Ferchaud-Roucher V et al. 2019; Venkatesh S. et al.2021; among others.
- CPT1c (b87498; Abcam, 1/1000) was already employed in Xiao G et al. 2013; Moon JS et al. 2016; Panahi M et al. 2020; among others.
- CPT2 (ab181114; Abcam, 1/1000) was already employed in Nomura M et al. 2016; Zhu J et al. 2019; Guo X et al. 2021; among others.
- GFAP (G6171; Sigma, 1/500) was already employed in Vinukonda G et al 2012; Tse KH et al 2014; among others.
- HSP60 (ab46798; Abcam, 1/1000) was already employed in Lee Y et al. 2021; Sánchez-Morán I et al. 2020; among others.
- NDUFS1 (sc-50132; Santa Cruz Biotechnology 1/500) was already employed in Martin MA et al. 2005; Duncan AM et al. 1992; among others.
- TOMM20 (ab56783; Abcam, 1/1000) was already employed in Darna M et al. 2009; Yarham JW et al. 2014; Zhang Y et al. 2020; Mu Y et al.2021; among others.
- UQCRC2 (ab14745; Abcam, 1/1000) was already employed in Kremer LS et al.2017; Zhao QY et al 2020; Chen C et al.2021; among others.
- B-Actin (A5441; Sigma 1/30000) was already employed in Melanie Si Yan Tan et al. 2019; Lorraine Springuel et al 2014; among others.
- B-Tubulin III (ab18207; Abcam 1/500) was already employed in Choi YS et al 2020; Navneet S et al 2019; among others.
- NDUFB8 (ab110242; Abcam; 1/1000) was already employed in Ghosh S et al. 2020; Hollinshead KER et al. 2020; among others.
- NDUFA9 (ab14713; Abcam, 1/1000) was already employed in Calvo E et al. 2020; González-García P et al. 2020; among others.
- SDHA (ab14715; Abcam, 1/1000) was already employed in Benegiamo G et al. 2022; Greggio C el al. 2017; among others.
- MTCO1 (ab14705; Abcam, 1/1000) was already employed in Balsa E et al. 2019; Greggio C el al. 2017; among others.
- COX IV (ab16056; Abcam, 1/1000) was already employed in Kontou G et al. 2021; Acoba MG et al. 2021; among others.
- IBA1 (019-19741; Wako, 1/1000) was already employed in Monai H et al. 2016; Sato M et al. 2020.; among others.
- MAP2 (ab32454; Abcam, 1/1000) was already employed in Müller A et al. 2015; Gonzales PK et. al 2018; among others.
- OLIG2 (ab109186; Abcam, 1/1000) was already employed in Bolaender A et al. 2021; Ravindra Kumar S et al. 2020; among others.
- PDH (3205; Cell Signaling, 1/1000) was already employed in Jiao Y et al. 2023; Stojakovic A et al. 2021; among others.
- pSer293-PDH (#31866; Cell Signaling, 1/1000) was already employed in Taylor SR et al. 2021; Stojakovic A et al. 2021; among others.
- GFP (ab290; Abcam, 1/5000) was already employed in Baumgartner P et al. 2018; Cerina M et al. 2020; among others.
- GFAP (G9269; Sigma, 1/500) was already employed in Honda M et al. 2017; Meyer LC et al. 2017; among others.

Animals and other research organisms

Policy information about studies involving animals; ARRIVE guidelines recommended for reporting animal research, and Sex and Gender in Research

Laboratory animals

Cpt1a(lox/lox) mice (PMID: 25830893) in C57BL6/J background were used. Animales were i.v. injected with adeno-associate virus

Laboratory animals	particles expressing Cre recombinase under cell-specific promoter to obtain animals with conditional reduction of carnitite palmitoyl transferase-1a. Male mice were used for in vivo experiments, from 2 to 12 months of age.
Wild animals	No wild animals were used in the study
Reporting on sex	Males were used for behavioral analysis as females were prioritized for mating (generation of primary astrocytes). Since no previous information is available on the impact of sex on CPT1A in astrocytes, we primarily used male mice for behavioral analyses to obtain proof of principle with the minimum possible number of animals. However, the key findings of our work, namely, the mitochondrial CI superassembly in supercomplexes in astrocytes upon CPT1A knockout, and the CI disassembly from supercomplexes in neurons from astrocytic-specific CPT1A knckout mice, were equally observed in males and in females.
Field-collected samples	No field collected samples were used in the study.
Ethics oversight	Procedures were approved by the Bioethics Committee of the University of Salamanca or CIC bioGUNE (PET and MRS) in accordance with the Spanish legislation (RD53/2013).

Note that full information on the approval of the study protocol must also be provided in the manuscript.

Flow Cytometry

Plots

Confirm that:

☒ The axis labels state the marker and fluorochrome used (e.g. CD4-FITC).

☒ The axis scales are clearly visible. Include numbers along axes only for bottom left plot of group (a 'group' is an analysis of identical markers).

☒ All plots are contour plots with outliers or pseudocolor plots.

☒ A numerical value for number of cells or percentage (with statistics) is provided.

Methodology

Sample preparation	Astrocytes and neurons acutely isolated immunomagnetically from adult mice brain were used. These cells were treated with the corresponding probes (Mitosox, DilC1),following manufacturer instructions, during a determined time. After incubation with the corresponding probe, the cells were centrifuged and the pellets were resuspended in PBS for further analysis.
Instrument	FACScalibur flow cytometer (BD Biosciences), equipped with a 15 mW argon laser
Software	CellQuest ™ v5 for adquisition and Paint-A-Gate ™ PRO (BD Biosciences) and FlowJo v10 for data quantification.
Cell population abundance	At least 100,000 events were acquired in triplicate and by condition.
Gating strategy	The threshold of the analyzer was adjusted in the corresponding channel of the flow cytometer to exclude most subcellular residues or cellular aggregates in the SSC/FSC plot. The median intensity values were obtained for each sample, and the FMO (unstained cells) substracted.

☒ Tick this box to confirm that a figure exemplifying the gating strategy is provided in the Supplementary Information.
