# Supplementary material for: Fatty acid oxidation organizes mitochondrial supercomplexes to sustain astrocytic ROS and cognition
Source: Nat Metab. 2023 Jul 17;5(8):1290–302. doi: 10.1038/s42255-023-00835-6 (PMC10447235; doi:10.1038/s42255-023-00835-6)
Supplement: Supplementary file 4 — This file contains a table with the names, source, catalogue number and references of the antibodies used. [file 42255_2023_835_MOESM4_ESM.docx]

**Antibody Dilution Species Commercial source Reference**

| **Primary antibodies for Western Blotting** | | | | |
| --- | --- | --- | --- | --- |
| **Β-Actin** | 1/30000 | Mouse | A5441, Sigma | Lorraine Springuel et al 2014; Melanie Si Yan Tan et al. 2019 |
| **CPT1a** | 1/1000 | Mouse | ab128568, Abcam | Horie T et al. 2012; Lounis MA et al. 2017; Wang L et al. 2021 |
| **CPT1b** | 1/1000 | Rabbit | ab134988, Abcam | Eleftheriadis T et al. 2016; Ferchaud-Roucher V et al. 2019; Venkatesh S et al. 2021 |
| **CPT1c** | 1/1000 | Rabbit | ab87498, Abcam | Xiao G et al. 2013; Moon JS et al. 2016; Panahi M et al. 2020 |
| **CPT2** | 1/1000 | Rabbit | ab181114, Abcam | Nomura M et al.2016; Zhu J et al. 2019; Guo X et al. 2021 |
| **TOMM20** | 1/1000 | Mouse | ab56783, Abcam | Darna M et al 2009; Yarham JW et al. 2014; Zhang Y et al. 2020; Mu Y et al. 2021 |
| **HSP60** | 1/1000 | Mouse | ab46798, Abcam | Sánchez-Morán I et al. 2020; Lee Y et al. 2021 |
| **GFAP** | 1/500 | Mouse | G6171, Sigma | Tse KH, et al 2014; Vinukonda G et al 2012 |
| **NDUFS1** | 1/500 | Goat | sc-50132, Santa Cruz | Duncan AM et al. 1992; Martin MA et al. 2005 |
| **UQCRC2** | 1/1000 | Mouse | ab14745, Abcam | Kremer LS et al. 2017;  Zhao QY et al 2020; Chen C et al. 2021 |
| **Β-Tubulin III** | 1/500 | Rabbit | ab18207, Abcam | Navneet S et al 2019; Choi YS et al 2020 |
| **NDUFB8** | 1/1000 | Mouse | ab110242, Abcam | Ghosh S et al. 2020; Hollinshead KER et al. 2020 |
| **NDUFA9** | 1/1000 | Mouse | ab14713, Abcam | Calvo E et al. 2020; González-García P et al. 2020 |
| **SDHA** | 1/1000 | Mouse | ab14715, Abcam | Benegiamo G et al. 2022; Greggio C el al. 2017 |
| **MTCO1** | 1/1000 | Mouse | ab14705, Abcam | Balsa E et al. 2019; Greggio C el al. 2017 |
| **COX IV** | 1/1000 | Rabbit | ab16056, Abcam | Kontou G et al. 2021; Acoba MG et al. 2021 |
| **IBA1** | 1/1000 | Rabbit | 019-19741; Wako | Monai H et al. 2016; Sato M et al. 2020 |
| **MAP2** | 1/1000 | Rabbit | ab32454; Abcam | Müller A et al. 2015; Gonzales PK et. al 2018 |
| **OLIG2** | 1/1000 | Rabbit | ab109186; Abcam | Bolaender A et al. 2021; Ravindra Kumar S et al. 2020 |
| **PDH** | 1/1000 | Rabbit | 3205; Cell Signaling | Jiao Y et al. 2023; Stojakovic A et al. 2021 |
| **pSer^293^-PDH** | 1/1000 | Rabbit | 31866; Cell Signaling | Taylor SR et al. 2021; Stojakovic A et al. 2021 |
| **Primary antibodies for inmunohistochemistry** | | | | |
| **GFP** | 1/5000 | Rabbit | ab290; Abcam | Milosevic A et al. 2016; Gong Q et al. 2021 |
| **Primary antibodies for flow citometry** | | | | |
| **CPT1a** | 1/500 | Mouse | ab128568, Abcam | Horie T et al. 2012; Lounis MA et al. 2017; Wang L et al. 2021 |
| **GFAP** | 1/500 | Rabbit | G9269, Sigma | Honda M et al. 2017; Meyer LC et al. 2017 |
| **Secondary antibodies for Western Blotting** | | | |  |
| **Mouse-HRP** | 1/10000 | Goat | 170-6516; Bio-Rad | Basu S et al. 2014; Hainer SJ et al, 2016; Yamamoto S et al. 2021 |
| **Rabbit-HRP** | 1/10000 | Goat | 170-6515; Bio-Rad | Bhat UG et al. 2015;  Zhu H et al. 2022 |
| **Goat-HRP** | 1/10000 | Rabbit | sc-2768; Santa Cruz | Zheng FQ et al. 2009;  Bruschetta G et al. 2018 |
| **Secondary antibodies for inmunohistochemistry** | | | | |
| **Rabbit-Cy2** | 1/500 | Goat | 111-225-144; Jackson Immunoresearch | Baumgartner P et al. 2018; Cerina M et al. 2020 |
| **Secondary antibodies for flow citometry** | | | | |
| **Rabbit-Cy2** | 1/500 | Goat | 111-225-144; Jackson Immunoresearch | Baumgartner P et al. 2018; Cerina M et al. 2020 |
| **Mouse-Cy5** | 1/500 | Goat | 115-175-003; Jackson Immunoresearch | Mazo C et al. 2022; Yan B et al. 2022 |

**Table RRID Identifiers.** Information on commercial sources and catalog numbers of antibodies.
