# Supplementary figures and images for: Fatty acid oxidation organizes mitochondrial supercomplexes to sustain astrocytic ROS and cognition
Source: Nat Metab. 2023 Jul 17;5(8):1290–302. doi: 10.1038/s42255-023-00835-6 (PMC10447235; doi:10.1038/s42255-023-00835-6)

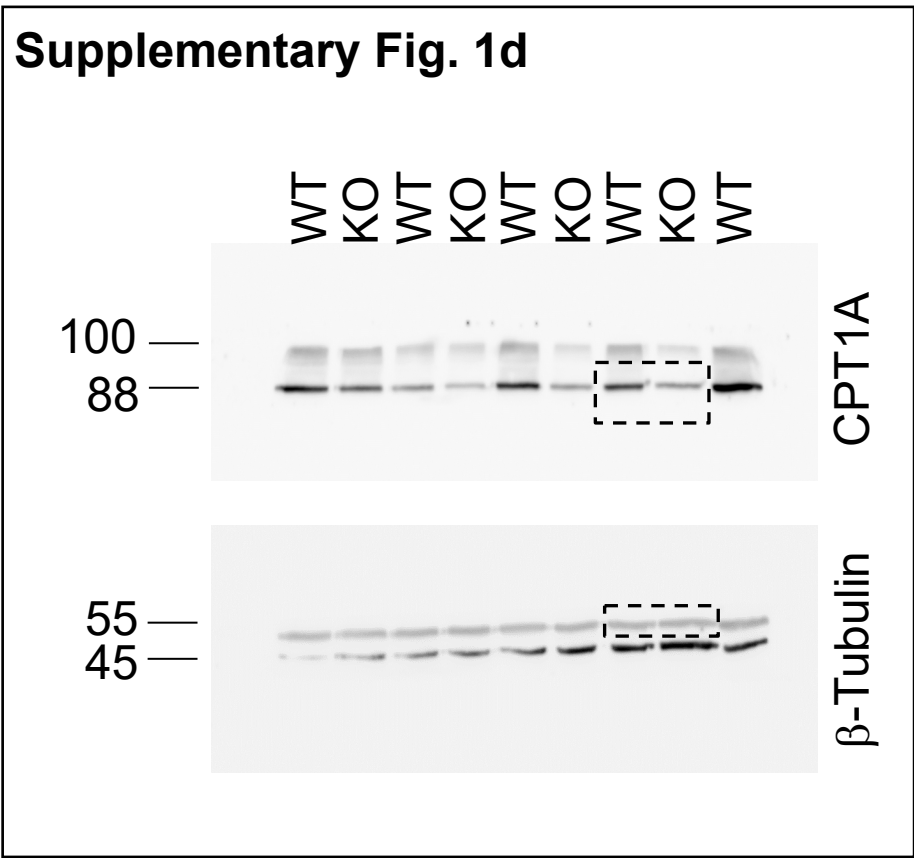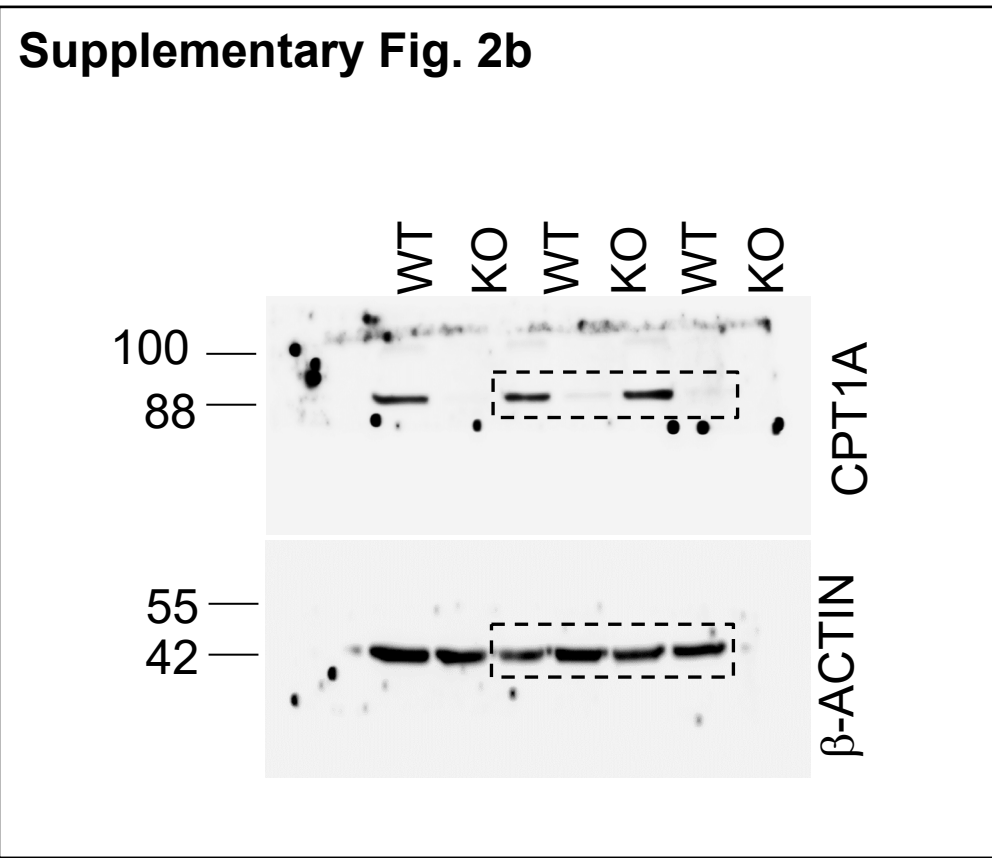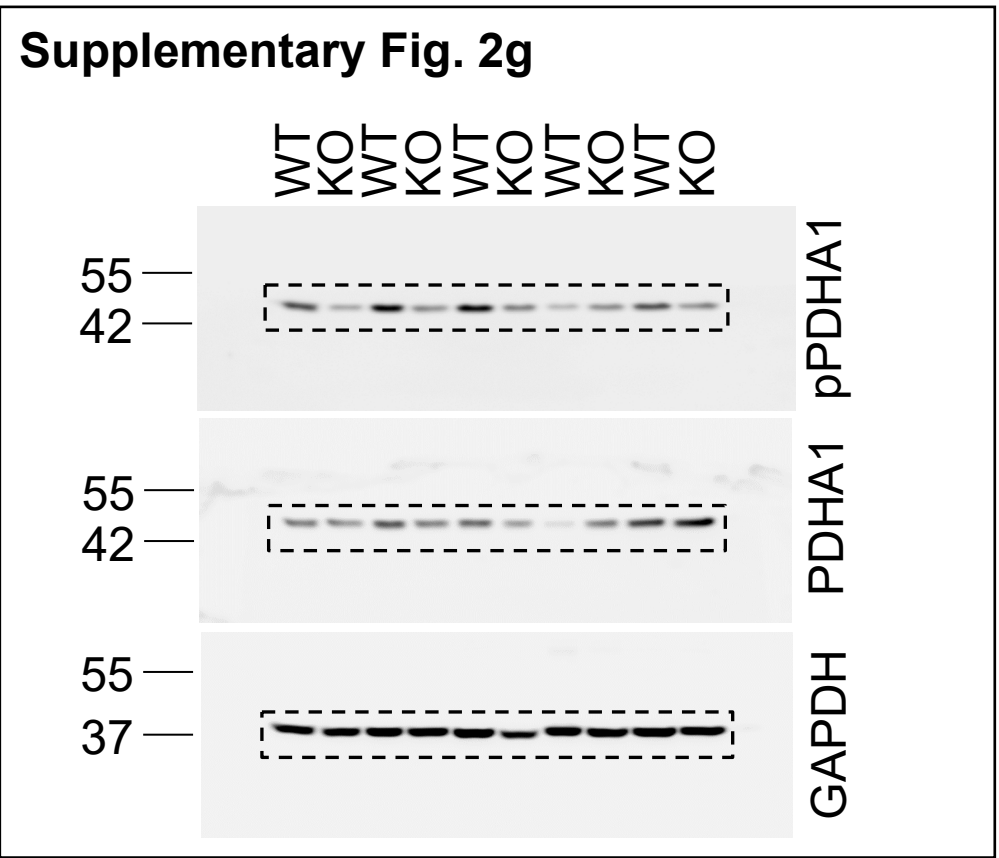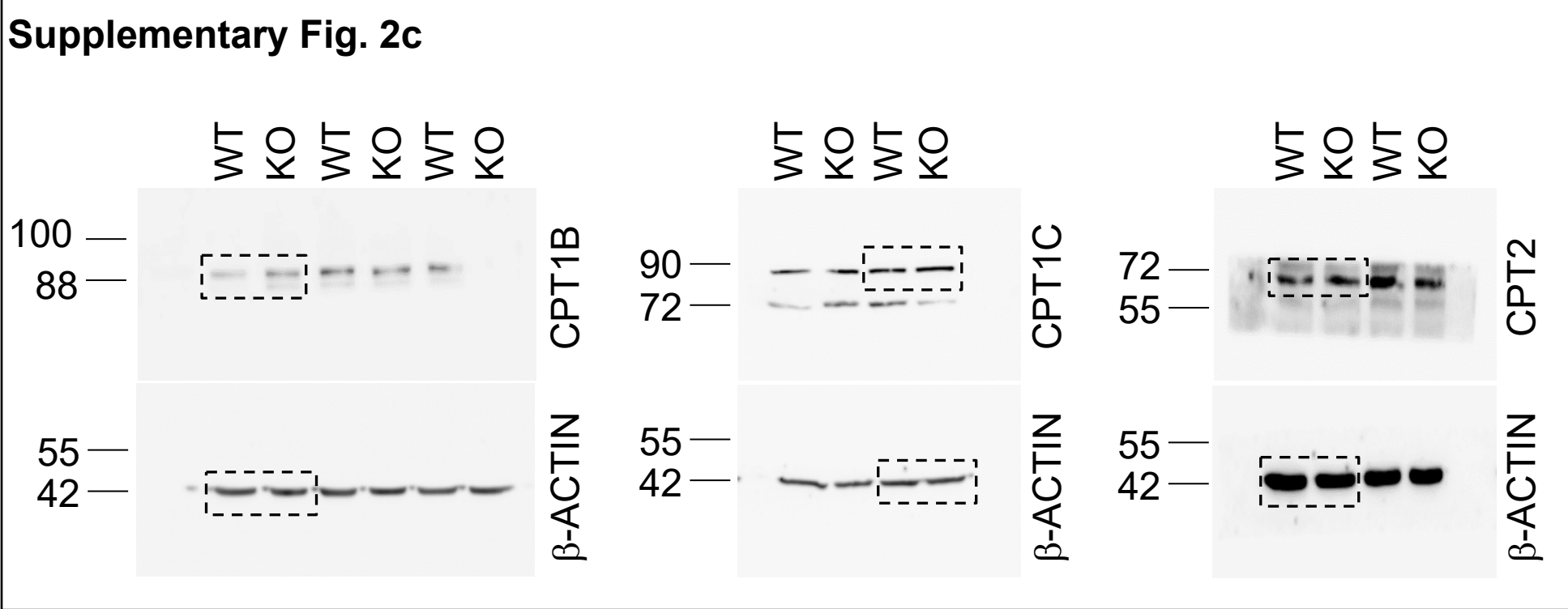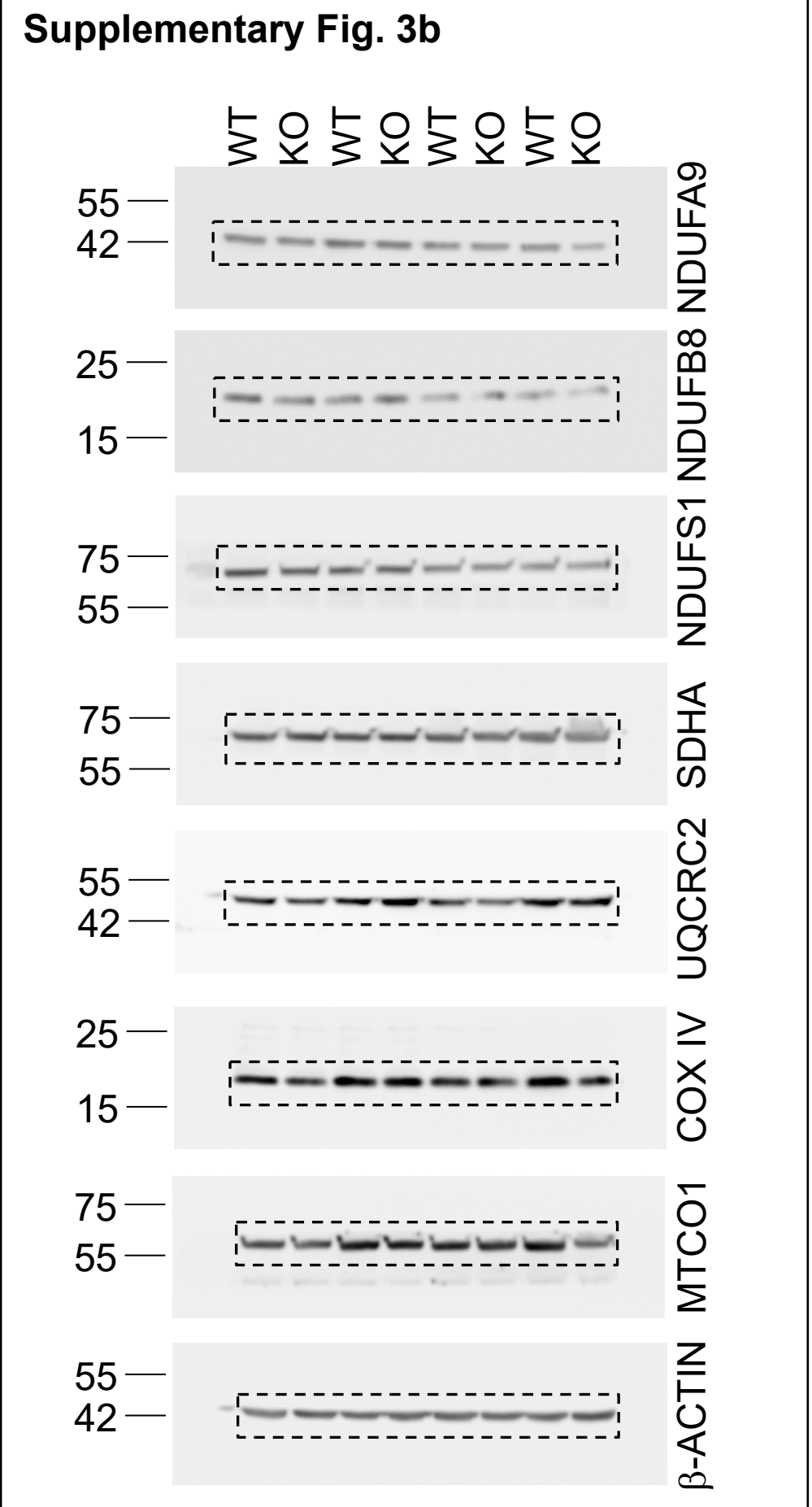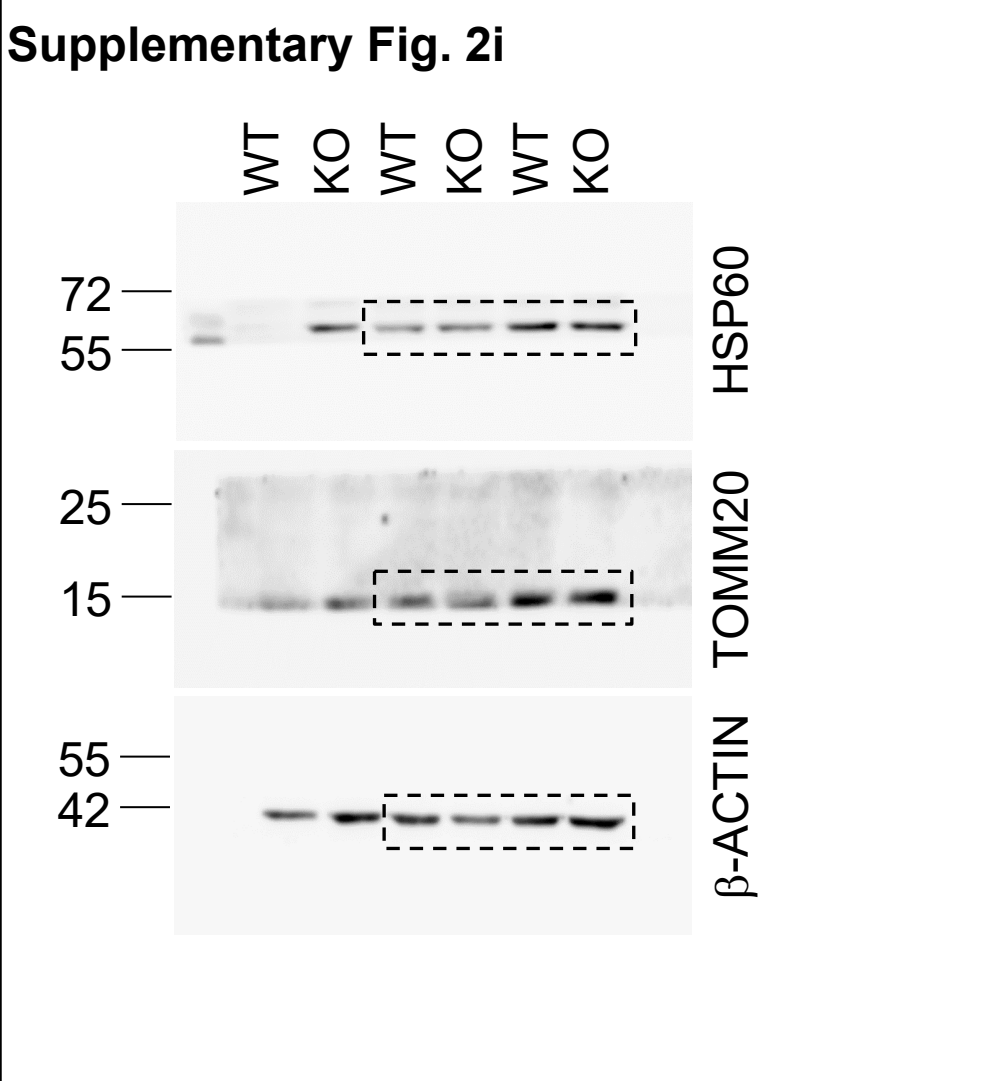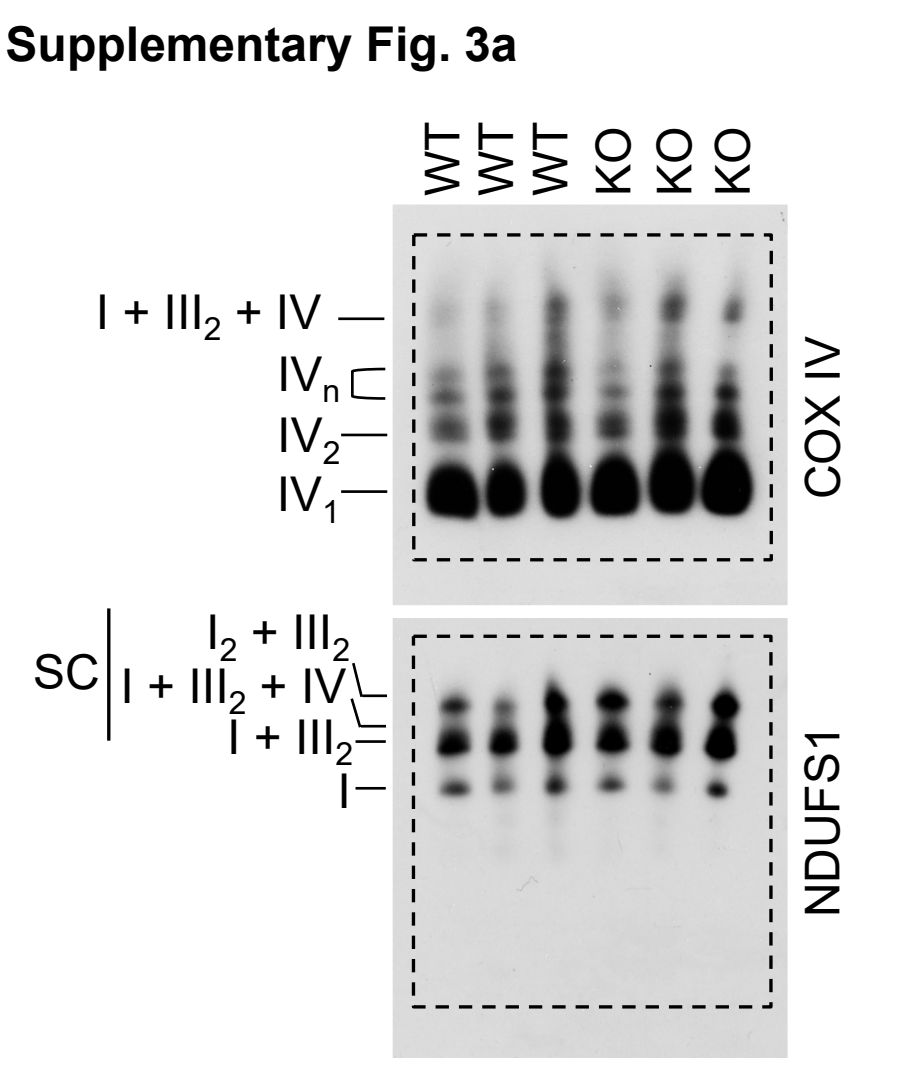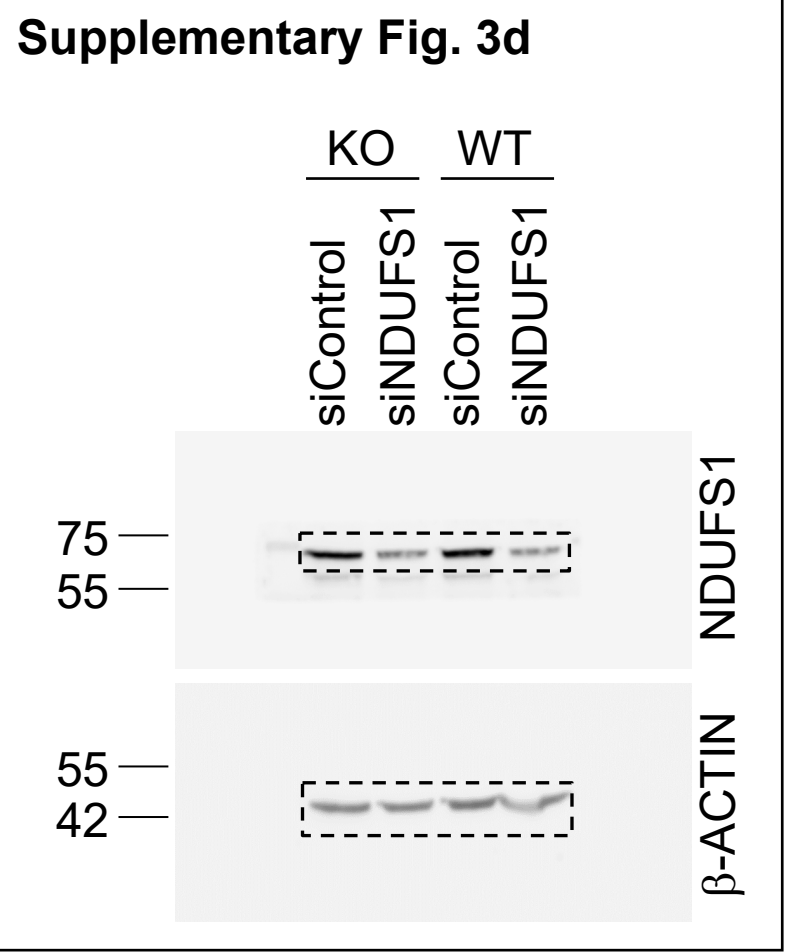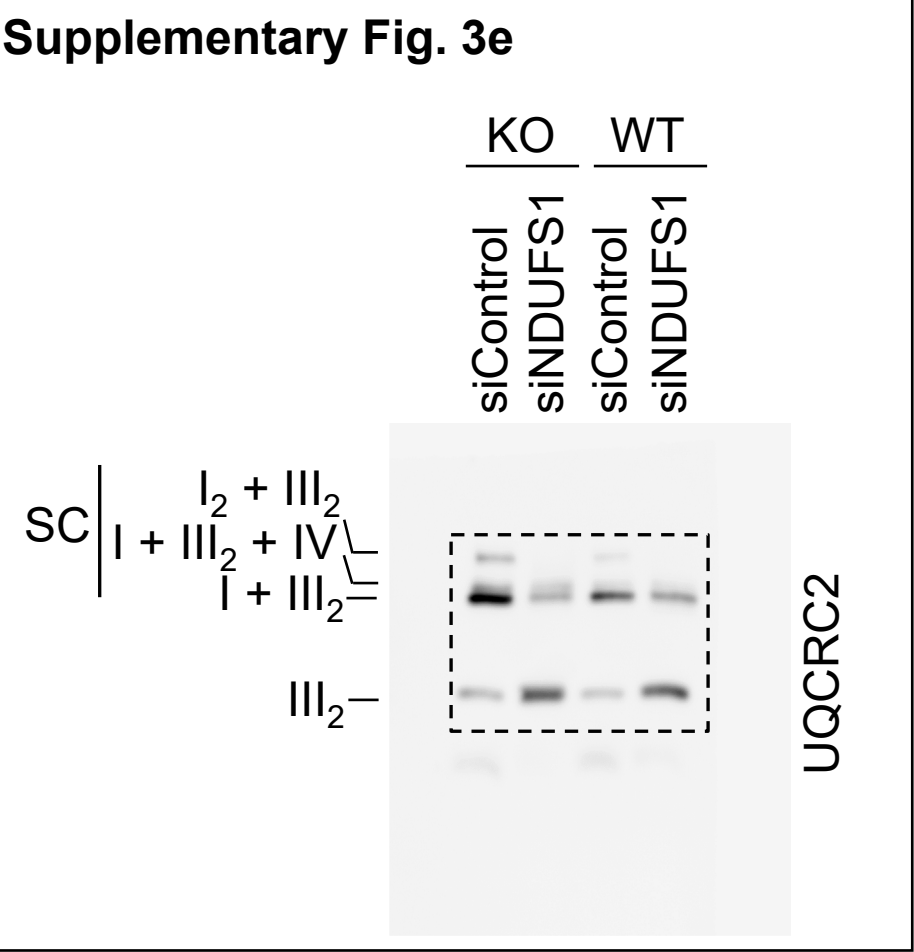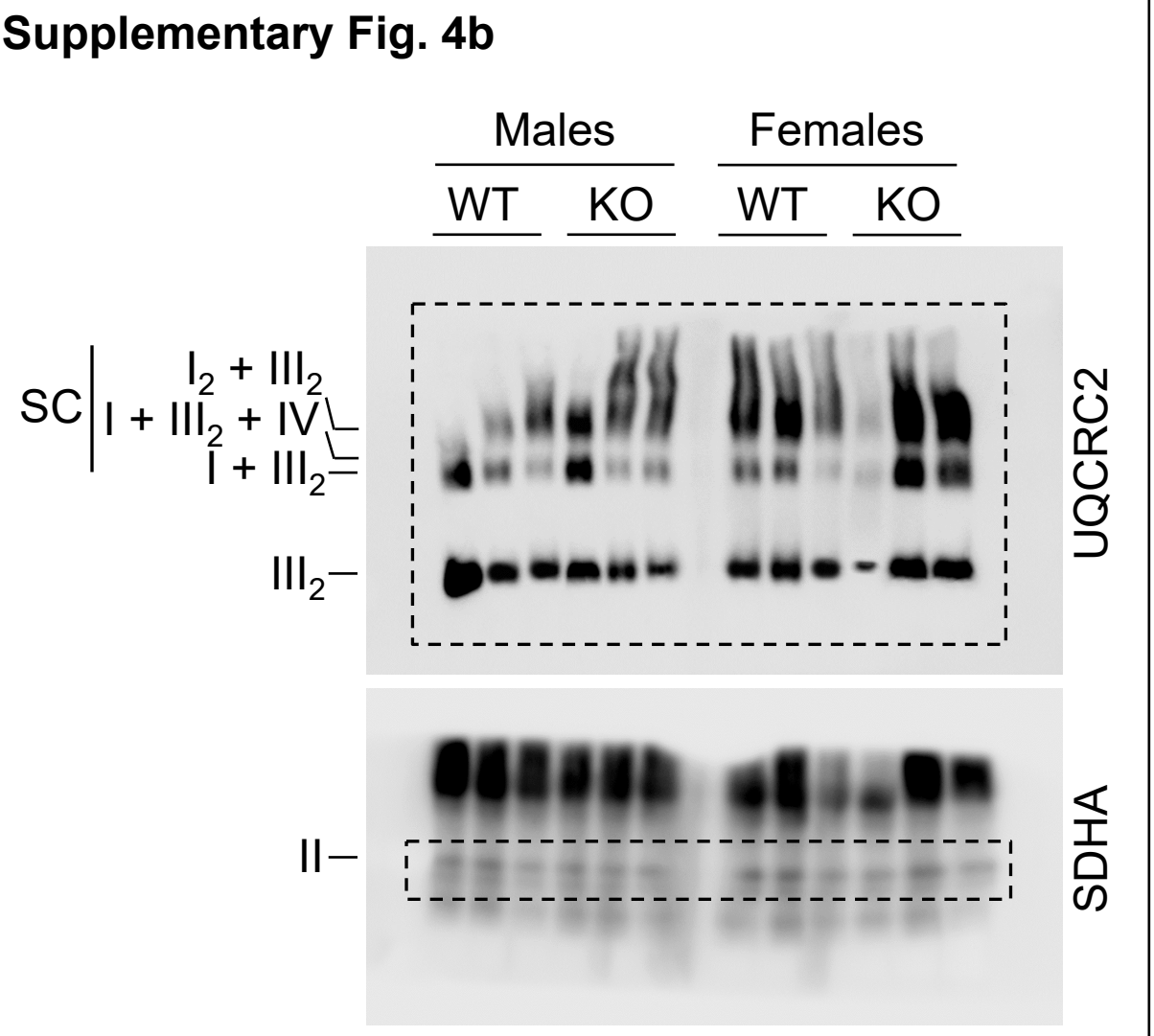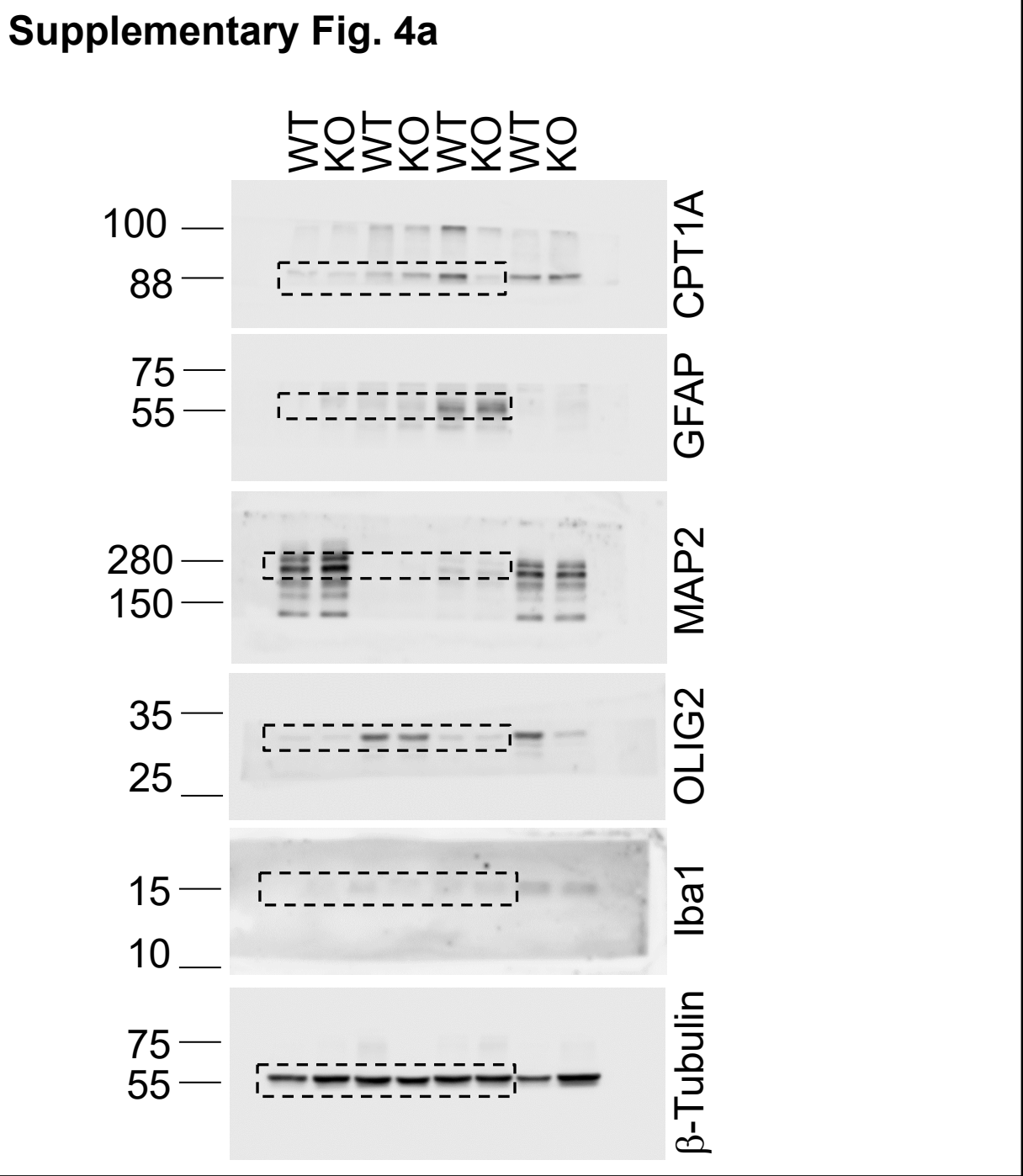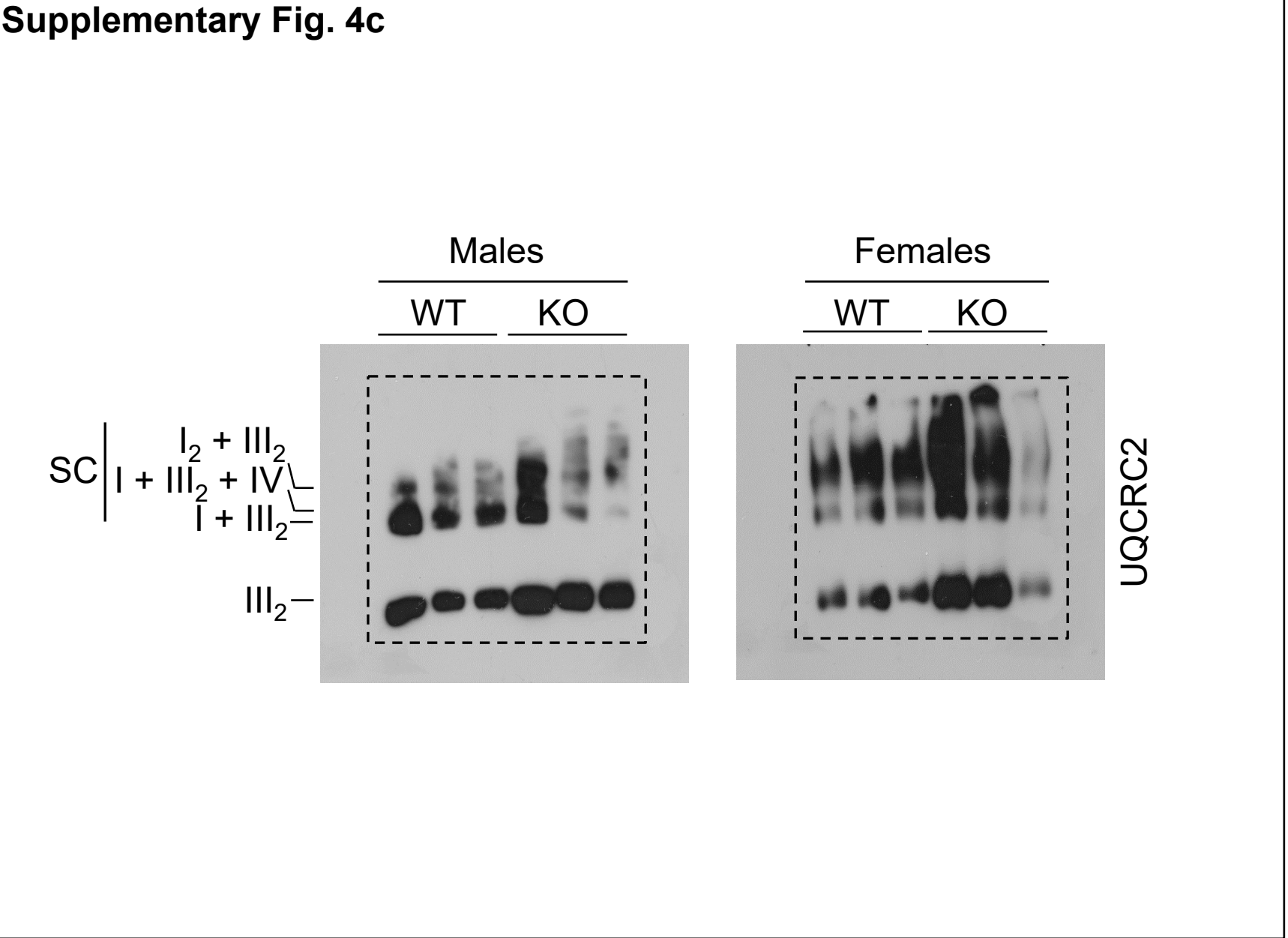

Supplement: Supplementary file 5 — This file contains the unprocessed and uncropped western blots shown in the supplementary information of the paper. [file 42255_2023_835_MOESM5_ESM.pdf]

**Fig. 1b**

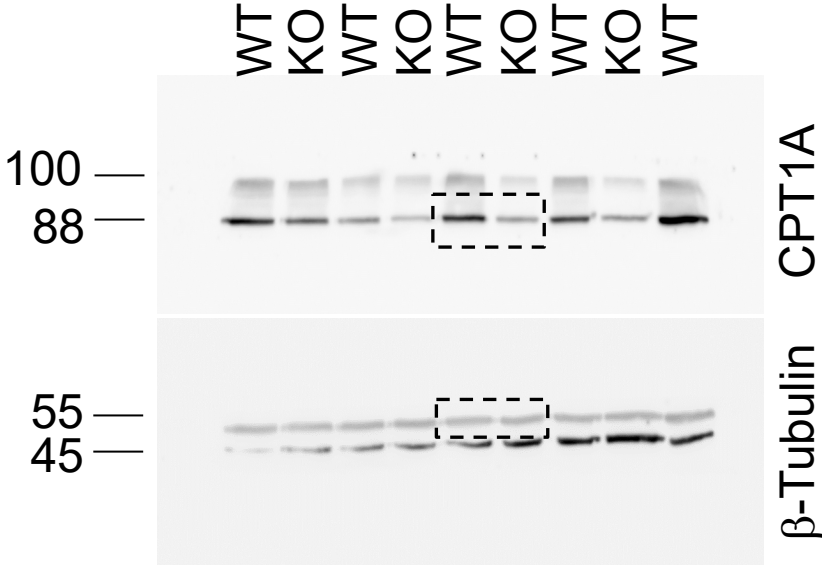

**Fig. 1c**

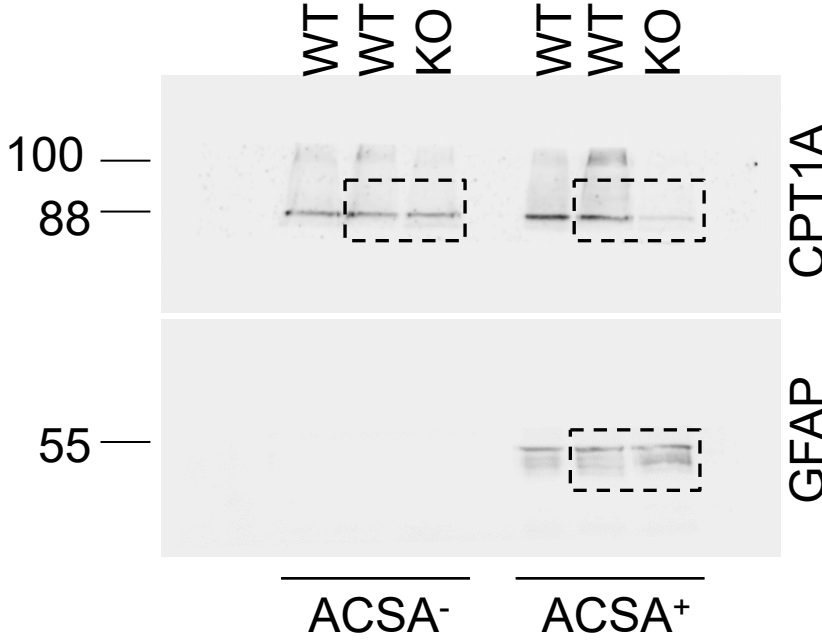

**Fig. 2b**

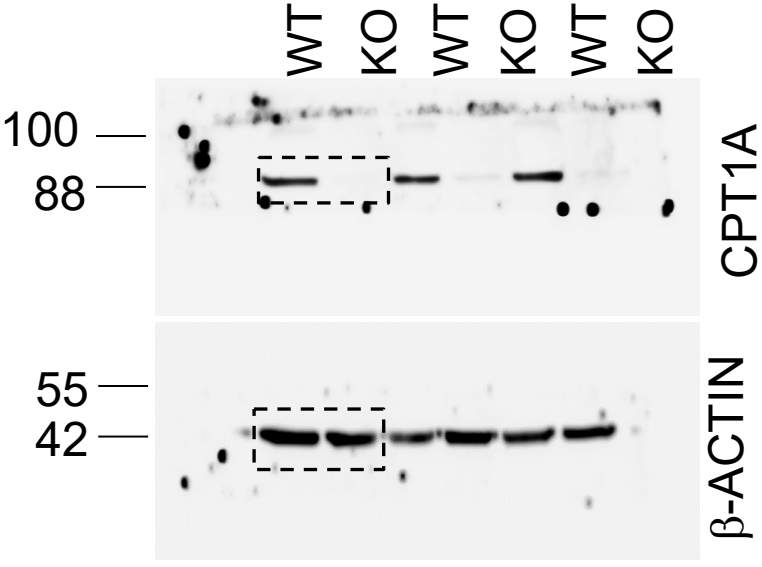

**Fig. 3a**

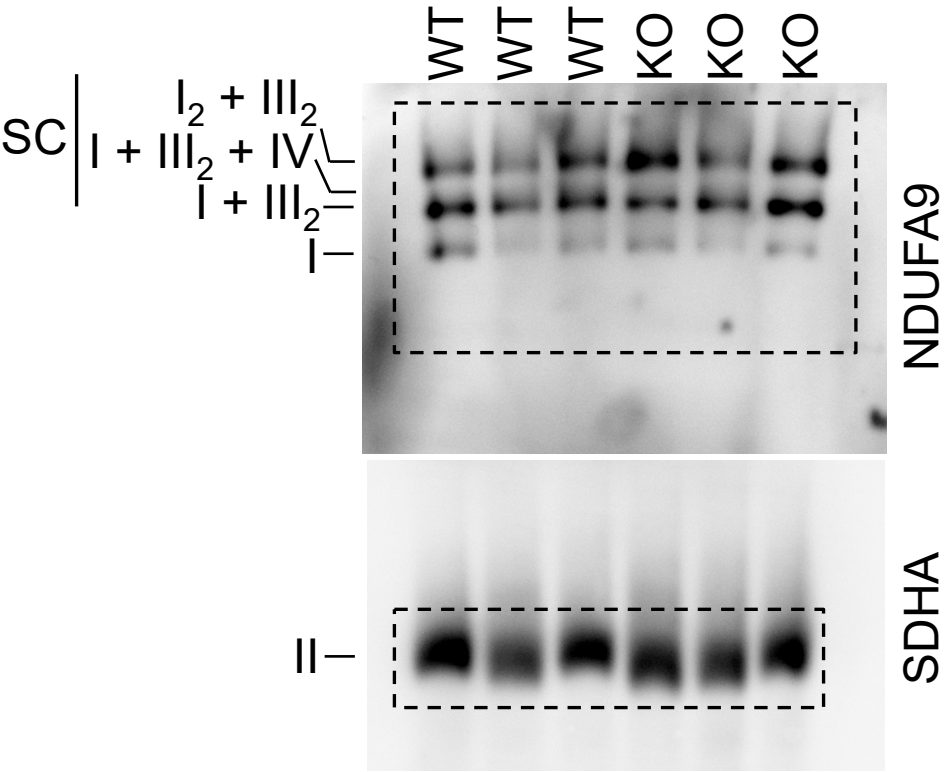

**Fig. 3b**

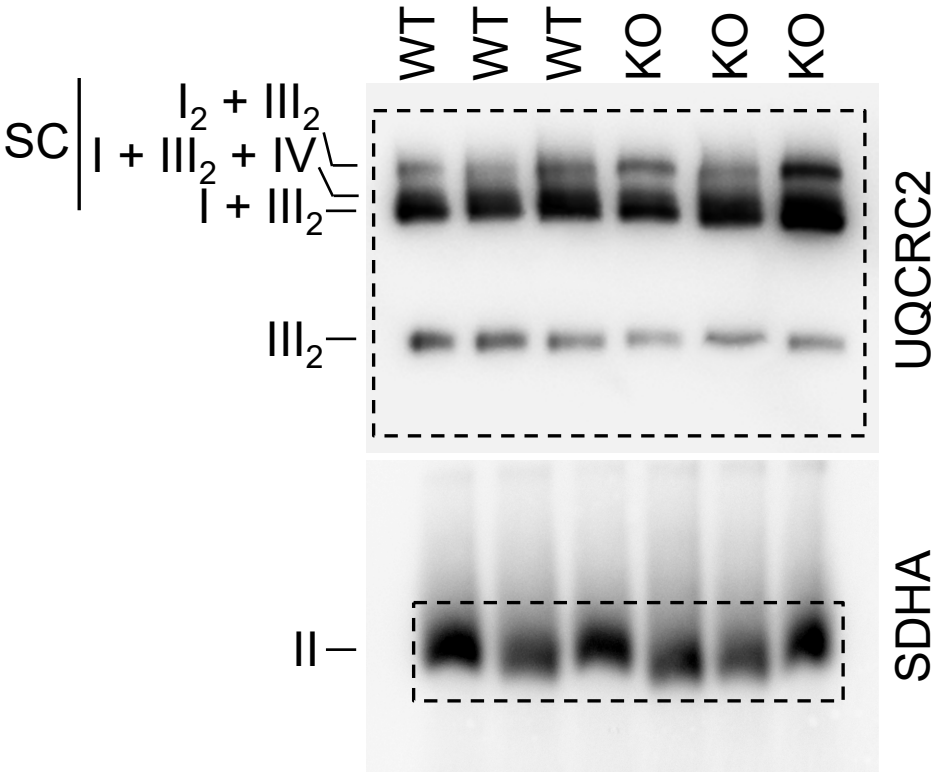

Supplement: Supplementary file 9 — This file contains the unprocessed and uncropped western blots shown in the main figures of the paper. [file 42255_2023_835_MOESM9_ESM.pdf]
