# Supplementary material for: Fatty acid oxidation organizes mitochondrial supercomplexes to sustain astrocytic ROS and cognition
Source: Nat Metab. 2023 Jul 17;5(8):1290–302. doi: 10.1038/s42255-023-00835-6 (PMC10447235; doi:10.1038/s42255-023-00835-6)
Supplement: Supplementary file 6 — This file contains representative plots of the flow cytometry analyses of the samples in the paper. [file 42255_2023_835_MOESM6_ESM.docx]

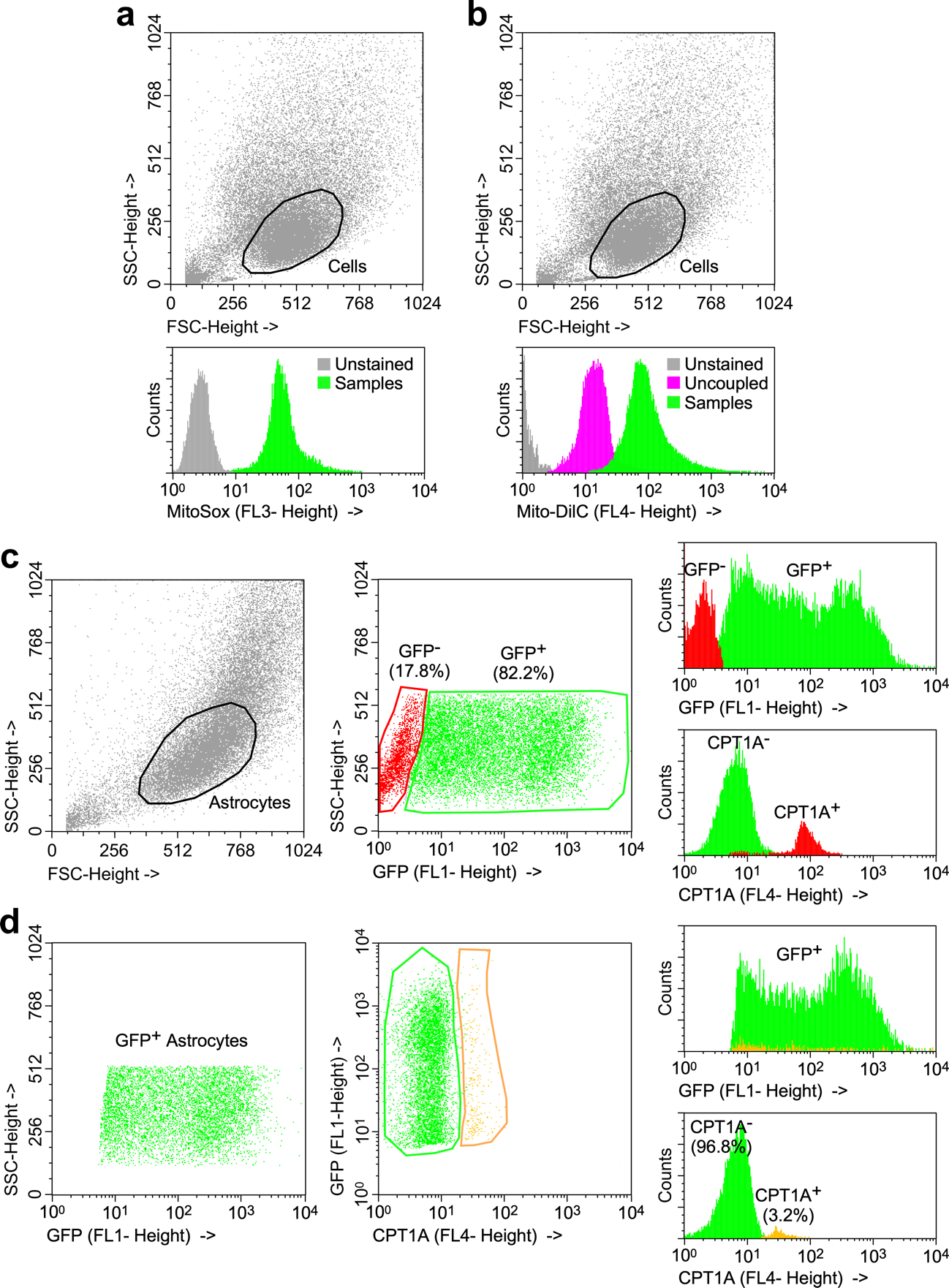


**Flow cytometry workflows.**

**(a)** MitoSox, to assess mitochondrial reactive oxygen species (mROS) in brain cells. The median of the selected population was used.

**(b)** DiIC1(5), to assess mitochondrial membrane potential (∆ψ_m_) in brain cells. ∆ψ_m_ is calculated subtracting, to the median of the selected population, the median of uncoupled population.

**(c)** GFP and CPT1A, to assess efficiency of transduction (GFP^+^ *versus* GFP^-^ cells) and the CPT1A-expressing cells (CPT1A^+^ *versus* CPT1A^-^ cells).

**(d)** GFP and CPT1A, to assess the proportion of efficiently transduced cells (GFP^+^) that express CPT1A (CPT1A^+^ cells).
